# Structure–Activity Relationship for the First-in-Class Clinical Steroid Sulfatase Inhibitor Irosustat (STX64, BN83495)

**DOI:** 10.1002/cmdc.201100288

**Published:** 2011-08-25

**Authors:** L W Lawrence Woo, Dharshini Ganeshapillai, Mark P Thomas, Oliver B Sutcliffe, Bindu Malini, Mary F Mahon, Atul Purohit, Barry V L Potter

**Affiliations:** [a] Medicinal Chemistry, Department of Pharmacy and Pharmacology, University of BathClaverton Down, Bath BA2 7AY (UK), Fax:(+44) 1225-386-114; [b] Diabetes, Endocrinology & Metabolism, Imperial College London, Section of Investigative Medicine6th Floor, Commonwealth Building (6N2B), Hammersmith Hospital, Du Cane Road, London W12 0NN (UK); [c] X-Ray Crystallographic Suite, Department of Chemistry, University of BathClaverton Down, Bath, BA2 7AY (UK)

**Keywords:** breast cancer, endocrine therapy, inhibitors, irosustat, steroid sulfatase, stx64

## Abstract

Structure–activity relationship studies were conducted on Irosustat (STX64, BN83495), the first steroid sulfatase (STS) inhibitor to enter diverse clinical trials for patients with advanced hormone-dependent cancer. The size of its aliphatic ring was expanded; its sulfamate group was N,N-dimethylated, relocated to another position and flanked by an adjacent methoxy group; and series of quinolin-2(1*H*)-one and quinoline derivatives of Irosustat were explored. The STS inhibitory activities of the synthesised compounds were assessed in a preparation of JEG-3 cells. Stepwise enlargement of the aliphatic ring from 7 to 11 members increases potency, although a further increase in ring size is detrimental. The best STS inhibitors in vitro had IC_50_ values between 0.015 and 0.025 nm. Other modifications made to Irosustat were found to either abolish or significantly weaken its activity. An azomethine adduct of Irosustat with *N*,*N*-dimethylformamide (DMF) was isolated, and crystal structures of Irosustat and this adduct were determined. Docking studies were conducted to explore the potential interactions between compounds and the active site of STS, and suggest a sulfamoyl group transfer to formylglycine 75 during the inactivation mechanism.

## Introduction

The inhibition of steroid sulfatase (STS) as a new target for endocrine therapy has attracted considerable attention over the past two decades after recognition that the STS pathway could also be a significant source of oestrogens alongside those originating from aromatase, the enzyme that aromatises androgens to oestrogens. Evidence to support this hypothesis includes: 1) a millionfold higher STS activity than aromatase activity in liver as well as normal and malignant breast tissues,[1] 2) the origin of oestrone (E1) from oestrone sulfate (E1S) in breast cancer tissue is ∼10-fold greater than that from androstenedione,[2] and 3) STS expression is an important prognostic factor in human breast carcinoma.[3, 4] Most oestrogens that originate from the aromatase pathway are converted into and stored in the body as sulfate conjugates that per se are biologically inactive. However, this reservoir of oestrogen sulfates could significantly contribute to overall oestrogenic stimulation of the growth and development of hormone-dependent tumours when STS catalyses the hydrolysis of substrates such as E1S to E1, and dehydroepiandrosterone sulfate (DHEA-S) to DHEA. The formation of DHEA via the STS pathway accounts for the production of 90 % of androstenediol (Adiol). Although structurally an androgen, Adiol possesses oestrogenic properties. It is ∼100-fold weaker than oestradiol[5–8] and has a lower affinity for the oestrogen receptor.[9] However, the 100-fold higher concentrations of Adiol in the circulation have led some to speculate that it may have oestrogenic properties equipotent to oestradiol.[10] Thus, STS is an attractive and novel target for rendering potentially more effective oestrogen deprivation through therapeutic intervention in hormone-dependent cancers such as those of the breast, endometrium, and prostate.

**Figure 1:**
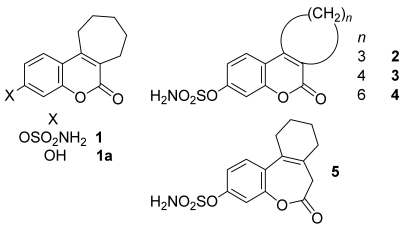
Structure of **1** (Irosustat, STX64), **1 a** (the parent phenol of **1**), and derivatives **2**–**5**.

Considerable progress has been made since the early 1990s in the development of STS inhibitors. Many structurally (steroidal and nonsteroidal) and mechanistically (principally reversible and irreversible) diverse inhibitors have been developed. However, compounds that contain the pharmacophore for irreversible inhibition of STS, i.e., an aryl sulfamate ester, have consistently shown distinctive and potent in vitro and in vivo inhibitory activities.[11–13] One compound, the nonsteroidal inhibitor **1** (Irosustat, STX64, BN83495, Figure 1), is the first STS inhibitor to enter clinical trials for postmenopausal patients with advanced hormone-dependent breast cancer and has shown encouraging results.[14, 15] Progress has been made since the completion of this first trial.[16] Currently, **1** is undergoing phase I trials for advanced prostate cancer and phase II trials for endometrial and advanced breast cancer.

On the discovery of **1** as a potent STS inhibitor, a basic study was carried out to provide a preliminary structure–activity relationship (SAR).[17] The main focus of that work was on ring contraction (from 7- down to 6- and 5-membered rings: compounds **2** and **3**, Figure 1) and expansion (from 7- to 8-membered rings: **4**, Figure 1) of the aliphatic ring of **1**. In addition, a tricyclic oxepin derivative of **3** (compound **5**, Figure 1) was synthesised and evaluated. Herein we report a more extensive SAR study for **1**, further expansion of the aliphatic ring size, N,N-dimethylation of the sulfamate group, relocation of the sulfamate group to another position, introduction of a substituent adjacent to the sulfamate group, and exploration of a series of quinolin-2(1*H*)-one and quinoline derivatives of **1**. The biological activities of the synthesised compounds were assessed in a preparation of JEG-3 cells. In addition, an azomethine adduct of **1** and *N*,*N*-dimethylformamide (DMF) is reported. The crystal structures of **1** and its azomethine adduct were determined. Docking studies were conducted to explore the potential interactions between the compounds and the active site of STS.

## Results and Discussion

### Chemistry

**Scheme 1:**
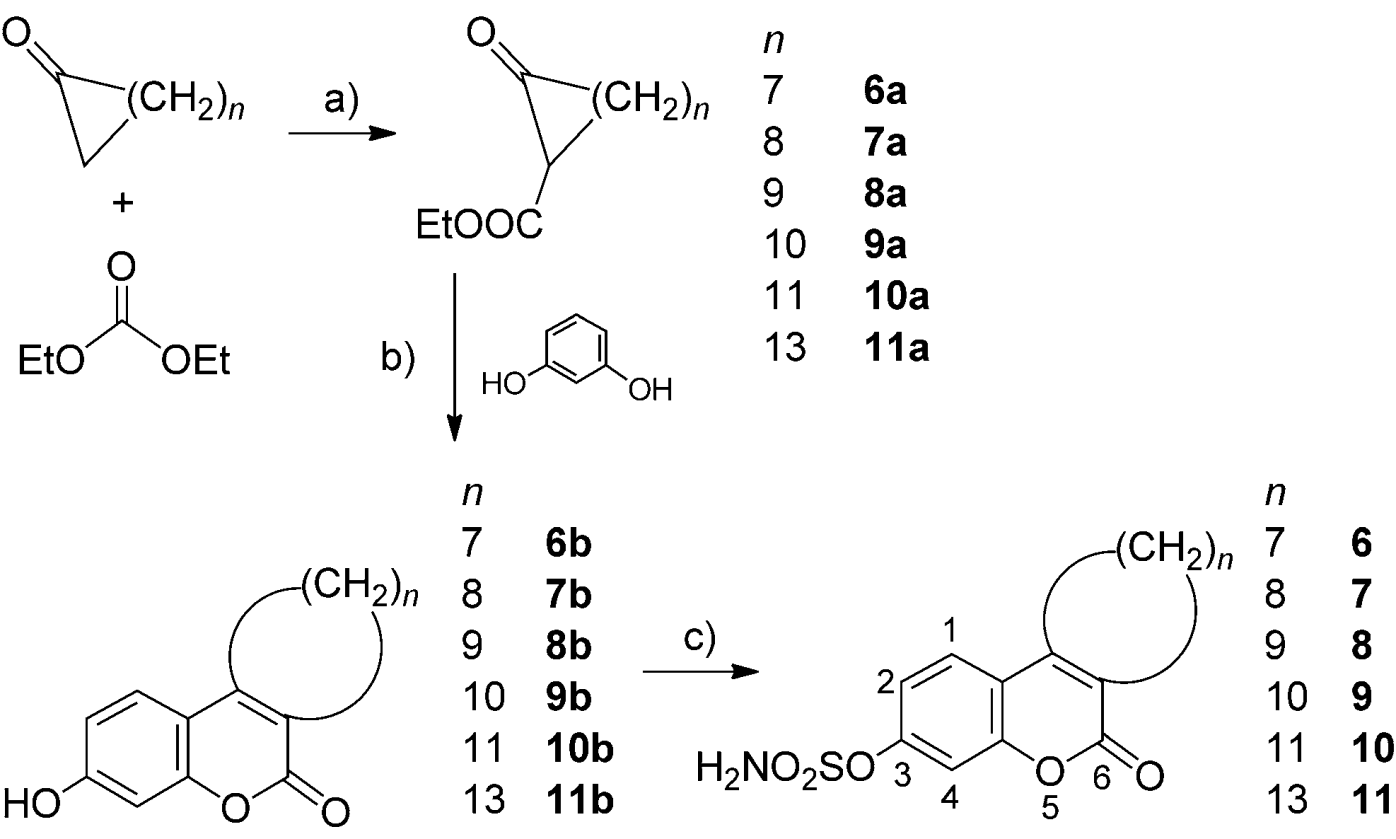
Synthesis of tricyclic coumarin sulfamates (**6**–**11**). *Reagents and conditions:* a) 2 NaH, N_2_, 15 h, RT; b) concd H_2_SO_4_/CF_3_COOH, 3 h, 0 °C→RT; c) anhydrous DMF, NaH, N_2_, H_2_NSO_2_Cl, 0 °C→RT.

With the exception of ethyl 2-oxocyclotridecanecarboxylate, which is available commercially, the starting cyclic β-keto esters required for the synthesis of tricyclic coumarins **6 b**–**9 b** and **11 b** were prepared by treating the corresponding cycloalkyl ketone with diethyl carbonate in the presence of two equivalents of sodium hydride at room temperature.[18] The parent tricyclic coumarins were formed under Pechmann conditions by cyclising resorcinol and the corresponding ethyl 2-oxocycloalkylcarboxylates in the presence of an equimolar mixture of trifluoroacetic acid and concentrated sulfuric acid as the condensing agent (Scheme 1). The yields of the tricyclic coumarins ranged from 14 to 33 %, presumably due to severe ring strain experienced by cycloalkenyl rings, in particular cyclononene and cycloundecene, during the cyclisation of the cyclic β-keto esters with resorcinol.

An earlier method was used for the sulfamoylation of parent hydroxycoumarins (Scheme 1). This involved treating a solution of the phenol in anhydrous *N*,*N*-dimethylformamide (DMF) with sodium hydride followed by the addition of a freshly concentrated solution of sulfamoyl chloride in toluene, which was prepared according to the method of Woo et al.[19]

**Scheme 2:**
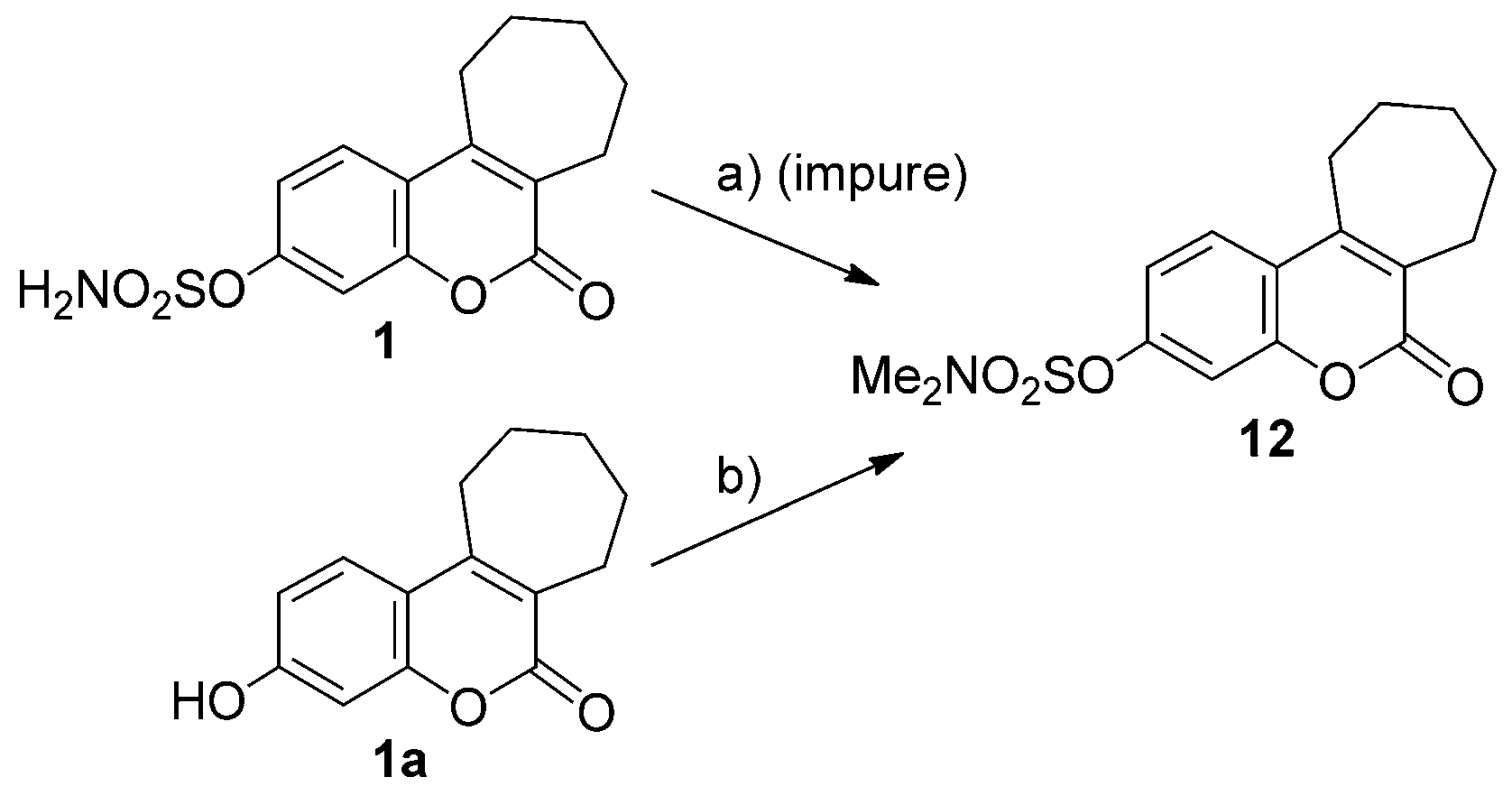
Synthesis of **12**, the *N*,*N*-dimethyl derivative of **1**. *Reagents and conditions:* a) NaH, CH_3_I, 0 °C (**12** obtained in this manner was contaminated by a trace amount of the 3-methoxy derivative of **1 a)**; b) *N*,*N*-dimethylcyclohexylamine, Me_2_NSO_2_Cl, 90–95 °C, 1 h.

The synthesis of **12** was initially attempted by deprotonation of **1** in *N*,*N*-dimethylacetamide (DMA) with sodium hydride at 0 °C followed by N,N-dimethylation with methyl iodide (Scheme 2). However, compound **12** obtained by this route was persistently contaminated by a trace amount of 3- methoxy-8,9,10,11-tetrahydrocyclohepta[*c*]chromen-6(7*H*)-one, which is most likely the product of desulfamoylation of **1** followed by methylation of the phenol released (compound **1 a**) under the reaction conditions employed. This ethereal contaminant was particularly difficult to remove, and hence a different synthetic approach was sought. Compound **12** was subsequently prepared with high purity by heating **1 a** in *N*,*N*-dimethylcyclohexylamine with *N*,*N*-dimethylsulfonyl chloride (Scheme 2).

**Scheme 3:**
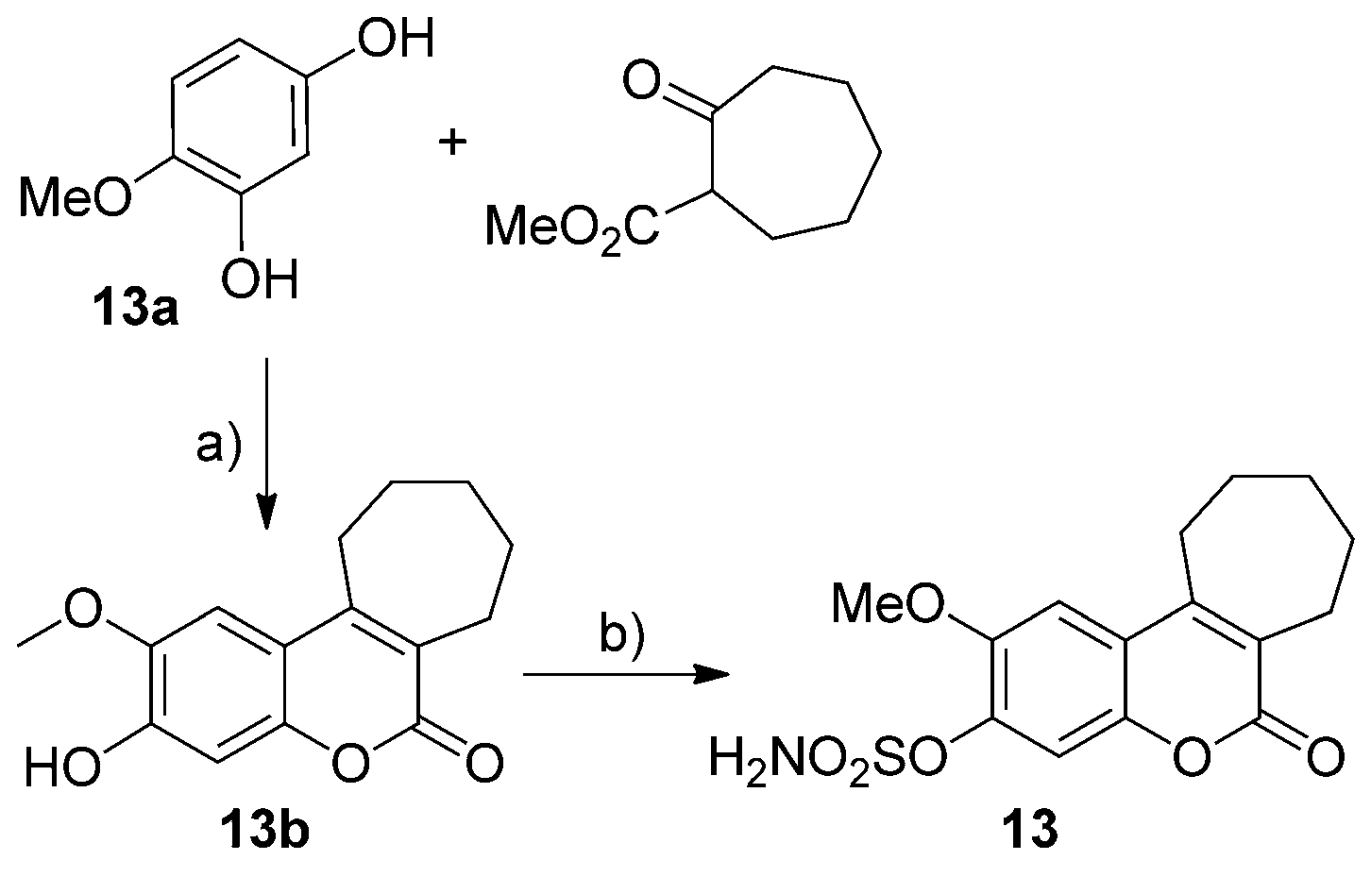
Synthesis of compound **13**. *Reagents and conditions:* a) concd H_2_SO_4_/CF_3_COOH, 0 °C→RT, 60 h; b) anhydrous DMA, N_2_, H_2_NSO_2_Cl, 0 °C→RT.

Similar to **1**, the synthesis of **13 b** was achieved by a Pechmann route, although resorcinol was replaced by 4-methoxybenzene-1,3-diol (**13 a**) as starting material, which was prepared according to the method of Godfrey et al. (Scheme 3).[20] Sulfamoylation of a solution of **13 b** in DMA gave the methoxylated tricyclic coumarin sulfamate **13**.

**Scheme 4:**
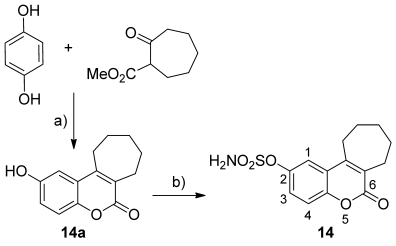
Synthesis of compound **14**. *Reagents and conditions:* a) concd H_2_SO_4_/CF_3_COOH, 0 °C→RT, 60 h; b) anhydrous DMA, N_2_, H_2_NSO_2_Cl, 0 °C→RT.

The synthesis of 2-hydroxy-8,9,10,11-tetrahydrocyclohepta[*c*]chromen-6(7*H*)-one (**14 a**) was carried out by allowing hydroquinone to react with methyl 2-oxo-1-cycloheptanecarboxylate under Pechmann conditions (Scheme 4). As anticipated, the isolated yield of **14 a** was extremely low (3 %) due to the 2-position of hydroquinone not being electron-rich and hence activated for ring closure by a Pechmann mechanism. Nonetheless, a sufficient quantity of **14 a** was isolated for further sulfamoylation to give the 2-sulfamate **14**.

**Scheme 5:**
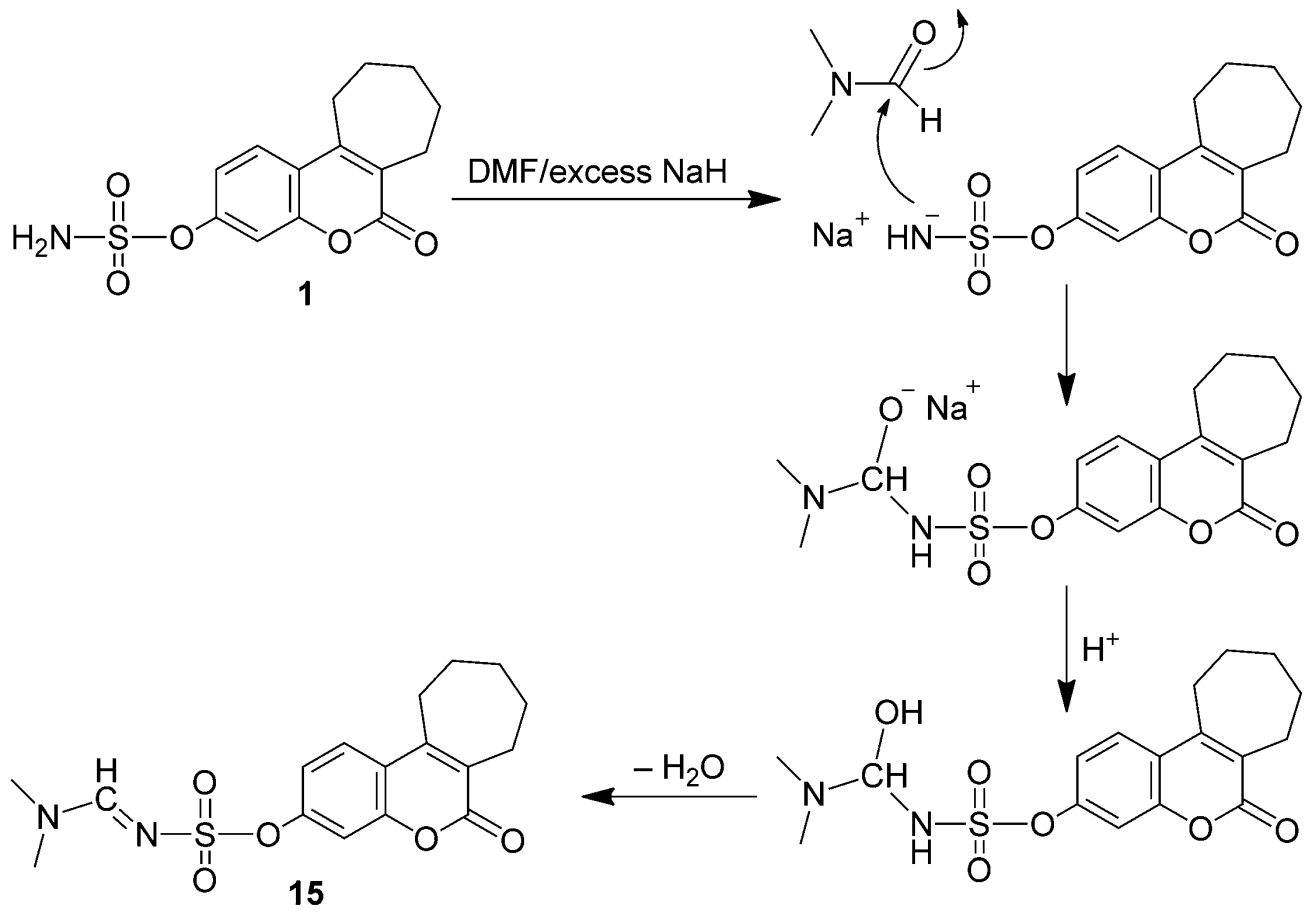
Proposed mechanism for the formation of **15**, an azomethine adduct between compound **1** and DMF.

Compound **15** is a low-yielding azomethine adduct of **1** with DMF. Only a very small amount of **15** was isolated during a very large-scale synthesis of **1** that was performed for determination of its crystal structure. With an earlier method for conducting sulfamoylation, which involves the use of sodium hydride in excess for deprotonating the phenolic parent compound **1 a** in DMF prior to the addition of sulfamoyl chloride, the formation of **15** is anticipated, as we reported earlier a similar azomethine adduct between 2-nitrophenyl sulfamate and DMF.[21] It is reasoned that the presence of excess sodium hydride in the reaction mixture deprotonates the sulfamate group of **1** after its formation, and the resulting anion undergoes a nucleophilic attack on the formyl group of DMF to give compound **15** upon subsequent dehydration, as illustrated in Scheme 5.

**Scheme 6:**
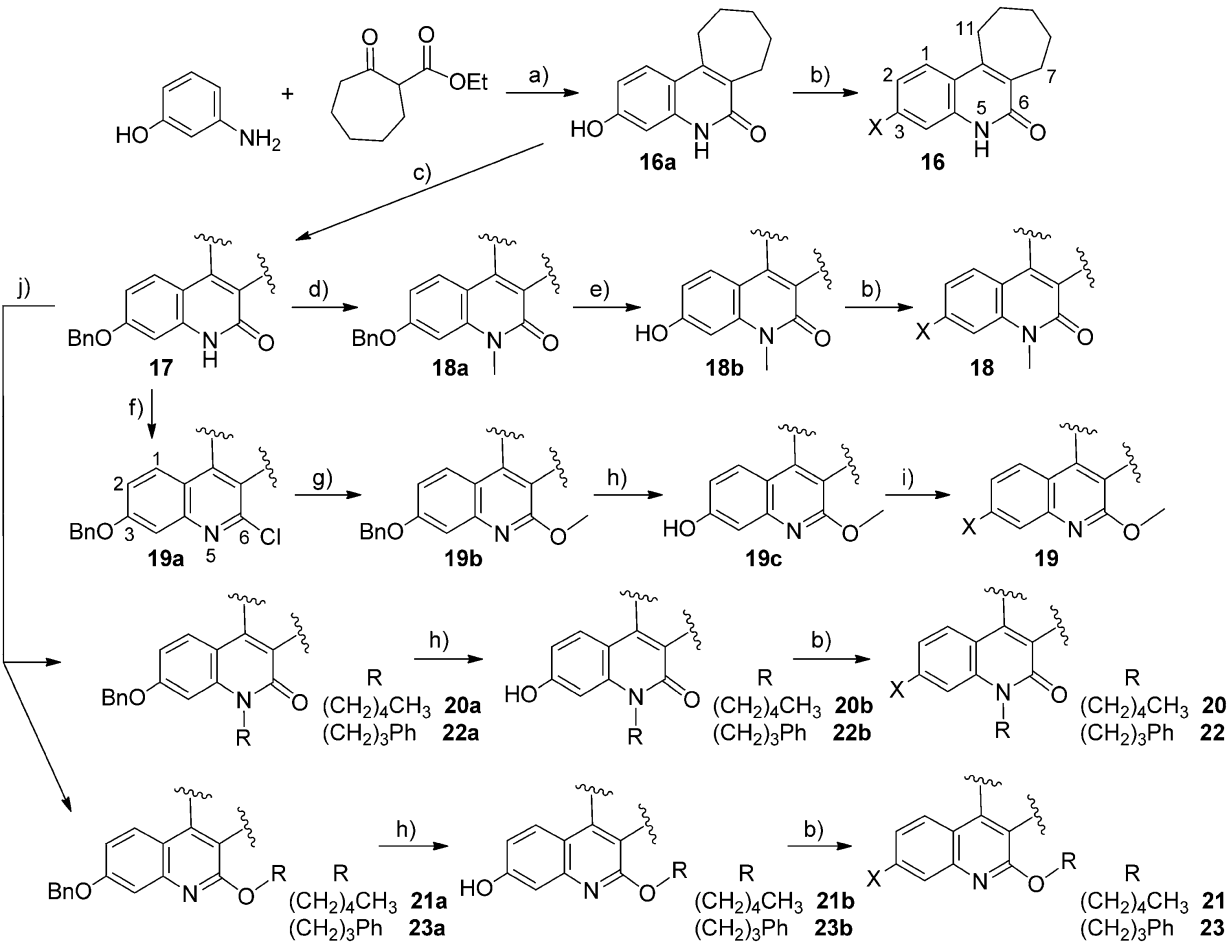
Synthesis of quinoline and quinolinone derivatives of **1**. *Reagents and conditions:* a) 150 °C, 8 h; b) anhydrous DMF, NaH, N_2_, H_2_NSO_2_Cl, 0 °C→RT; c) NaH, DMF, 0 °C, BnBr, 90 °C; d) NaH, DMF, 0 °C, CH_3_I, 80 °C; e) Pd/C (10 %), THF, H_2_ (balloon); f) POCl_3_, reflux; g) anhydrous DMF, NaH, anhydrous MeOH/DMF, 70 °C, 2 h; h) Pd/C (10 %), abs. EtOH, H_2_ (balloon); i) 2,6-di-*tert*-butyl-4-methylpyridine, N_2_, anhydrous CH_2_Cl_2_, H_2_NSO_2_Cl, RT; j) NaH, anhydrous DMF, 1-bromopentane or 1-bromo-3-phenylpropane, 100 °C, 1 h [X=OSO_2_NH_2_].

The quinolinone derivative **16 a** was prepared in good yield (73 %) by heating a mixture of 3-aminophenol and methyl 3-oxo-1-cycloheptane carboxylate (Scheme 6). Sulfamoylation of **16 a** in the usual manner gave the quinolinone sulfamate **16**.

The key intermediate for synthesising the rest of the quinoline and quinolinone derivatives reported herein is compound **17**, which was prepared by *O*-benzyl protection of **16 a** (Scheme 6). After deprotonation of **17** with sodium hydride and heating the resulting anion with methyl iodide, the *N*-methyl derivative **18 a** was obtained in high yield. Debenzylation by hydrogenation gave the phenolic quinolinone **18 b**, which was sulfamoylated to give the 5-methyl quinolinone sulfamate **18**.

The 3-*O*-benzyl-protected quinolinone **17** was converted into the 6-chloroquinoline **19 a** with phosphorus oxychloride. Holding **19 a** at reflux in anhydrous DMF with freshly prepared sodium methoxide gave the 6-methoxyquinoline **19 b**. The 6-methylquinolinyl sulfamate **19** was obtained by first debenzylating **19 b** followed by sulfamoylating the phenolic derivative **19 c**.

Quinolinones **20** and **22** and quinolines **21** and **23** were prepared by a different route from their corresponding lower members **18** and **19**. Holding the anion of **17** at reflux in DMF with either 1-bromopentane or 1-bromo-3-phenylpropane rendered a mixture of both the N- (**20 a** and **22 a**) and O-alkylated (**21 a** and **23 a**) derivatives. Interestingly, the isolated yields of quinolinones **20 a** (62 %) and **22 a** (55 %) were both found to be higher than their quinoline counterparts **21 a** (41 %) and **23 a** (42 %), suggesting that N-alkylation is slightly more favourable under the reaction conditions. In addition, both quinolinones were retained longer by silica in flash chromatography than quinolines, suggesting that **20 a** and **22 a** are more polar than **21 a** and **23 a**. Debenzylation by hydrogenation of **20 a**–**23 a** in the usual manner gave the phenolic derivatives **20 b**–**23 b**, which upon sulfamoylation gave the corresponding sulfamates **20**–**23**.

**Scheme 7:**
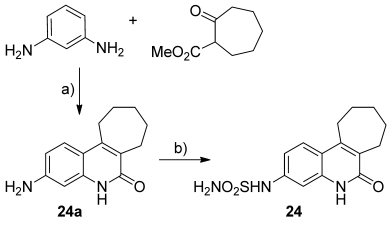
Synthesis of compound **24**. *Reagents and conditions:* a) 150 °C, 18 h; b) anhydrous DMF, N_2_, DBMP, H_2_NSO_2_Cl, 0 °C→RT.

The aminoquinolinone **24 a** was prepared by heating a mixture of 1,3-phenylenediamine and methyl 2-oxo-1-cycloheptane carboxylate at 150 °C overnight (Scheme 7). Upon sulfamoylation of a solution of **24 a** in DMF in the presence of 2,6-di-*tert*-butyl-4-methylpyridine (DBMP) and sulfamoyl chloride gave the sulfamido quinolinone **24**.

### Crystal structures

**Figure 2:**
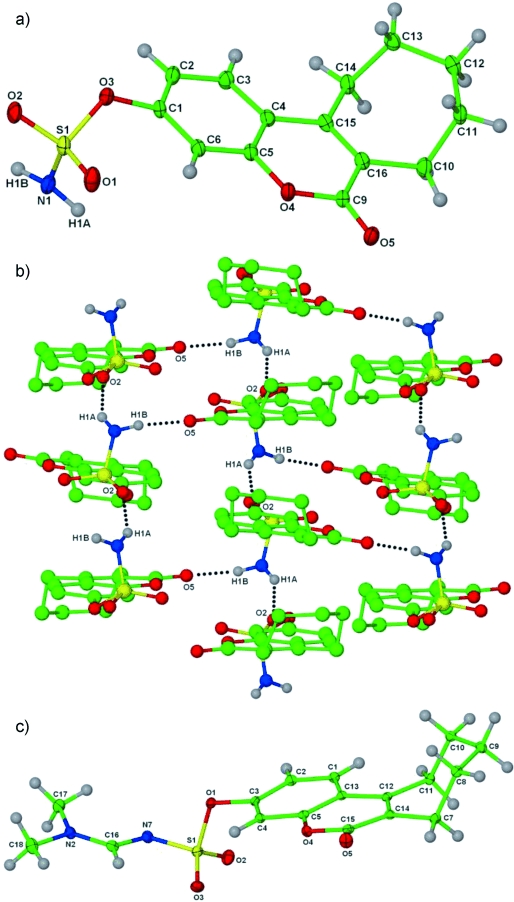
a) X-ray crystal structure of **1** (CCDC deposition code: 826524); ellipsoids are represented at 30 % probability. b) Portion of extended structure present in **1** showing the network of intermolecular hydrogen bonding. c) X-ray crystal structure of **15** (CCDC deposition code: 826525); ellipsoids are represented at 30 % probability.

A crystal of **1** with approximate dimensions of 0.25×0.10×0.08 mm was used for data collection. As shown in Figure 2 b, molecules of **1** interact via a network of intermolecular hydrogen bonds. In particular, one proton of the sulfamate NH_2_ group (H1B) interacts with the carbonyl oxygen atom (O5) of the coumarin ring in a proximate molecule, whereas the other NH proton (H1A) interacts with an oxygen atom (O2) of the SO_2_ group of a neighbouring sulfamate group. Additionally, there are possible intermolecular π–π interactions present (centroid_C9-C10-C15-C16_ to centroid_C1-C2-C3-C4-C5-C6_ distance=3.52 Å). As predicted in previous work by molecular modelling, the 7-membered aliphatic ring of **1** is in the chair form (Figure 2 a,b), which is similar to that of cycloheptene with the C=C moiety taking the place of one of the ring carbon atoms in the cyclohexane chair.[17]

A crystal of **15** with approximate dimensions of 0.25×0.13×0.10 mm was used for data collection. As shown in Figure 2 c, the tricyclic coumarin scaffold of **15** has a similar conformation to that observed for **1**. The stereochemistry is unambiguously *E* at the double bond of its (dimethylamino)methylene sulfamoyl group, suggesting that steric effects might be a contributing factor in the more favourable formation of the *trans* geometric isomer via the route in Scheme 5, with the bulky dimethylamino and arylsulfamoyl motifs placed diametrically opposite before the antiperiplanar elimination of water. As for **1**, the aliphatic ring of **15** is clearly in the chair form. Crystal structures of two other tricyclic coumarin sulfamates **6** and **7** with larger ring sizes were also obtained and have been reported elsewhere.[22]

### Structure–activity relationship and molecular modelling

Altogether, ten tricyclic coumarin sulfamates are compared in this work, out of which the syntheses of six final compounds are reported for the first time. These compounds contain a core bicyclic coumarin ring system, but differ in the size of the third (aliphatic) ring. The lowest member of the series studied is **2**, because having an aliphatic ring smaller than the 5-membered cyclopentenyl would be synthetically challenging due to the significant ring strain of a cyclobutene or cyclopropene. The increase in size of the third ring was carried out in a stepwise fashion from 5 to 15 members, although the 14-membered derivative was omitted, primarily due to the lack of commercial availability of cyclotetradecanone as starting material.

We evaluated the STS inhibitory activities of the tricyclic coumarin sulfamates **1**–**4** and **6**–**11** in a placental microsome preparation, and the results were reported in a previous publication.[23] For reference and comparison, these results are listed in Table 1. In this assay, **7** (10-membered third ring) proved to be the most potent STS inhibitor of the series in vitro, with an IC_50_ value of 1 nm, although **1** (7-membered third ring), **6** (9-membered third ring), and **8** (11-membered third ring) were also potent, with IC_50_ values ranging from 8 to 13 nm. The least potent congeners of the series were **2** (5-membered third ring) and **11** (15-membered third ring), the IC_50_ values for which were found to be 200 nm or higher. While it is not clear why the IC_50_ value for **4** (8-membered third ring) is not of the same order of magnitude as its immediate lower (**1**) and higher (**6**) congeners, but is instead significantly higher at 30 nm, it is apparent that the size of the third ring in this series of compounds has a marked effect on the potency of compounds against STS. Interestingly, it was found that **7** is only marginally more potent than **1** in vivo despite its IC_50_ value in placental microsomes at 1 nm being eightfold lower than that of **1**.[23] Despite its relatively weak activity in vitro (IC_50_=370 nm, placental microsomes), **11** was found to be the most potent tricyclic coumarin sulfamate in vivo, inhibiting rat liver STS activity by 23 and 94 % when assayed 24 h after administration at respective doses of 0.1 and 1 mg kg^−1^,[23] which may be explained, among other things, by a depot effect relating to its high log *P* value.

**Table 1:** Inhibition of STS activity in placental microsomes (PM) and JEG-3 cells by tricyclic coumarin sulfamates **1**–**4** and **6**–**11**, the *N*,*N*-dimethyl derivative of **1** (compound **12**), the 2-methoxy derivative of **1** (**13**), 6-oxo-6,7,8,9,10,11-hexahydrocyclohepta[*c*]chromen-2-yl sulfamate (**14**), and the azomethine adduct of **1** and DMF (**15**)

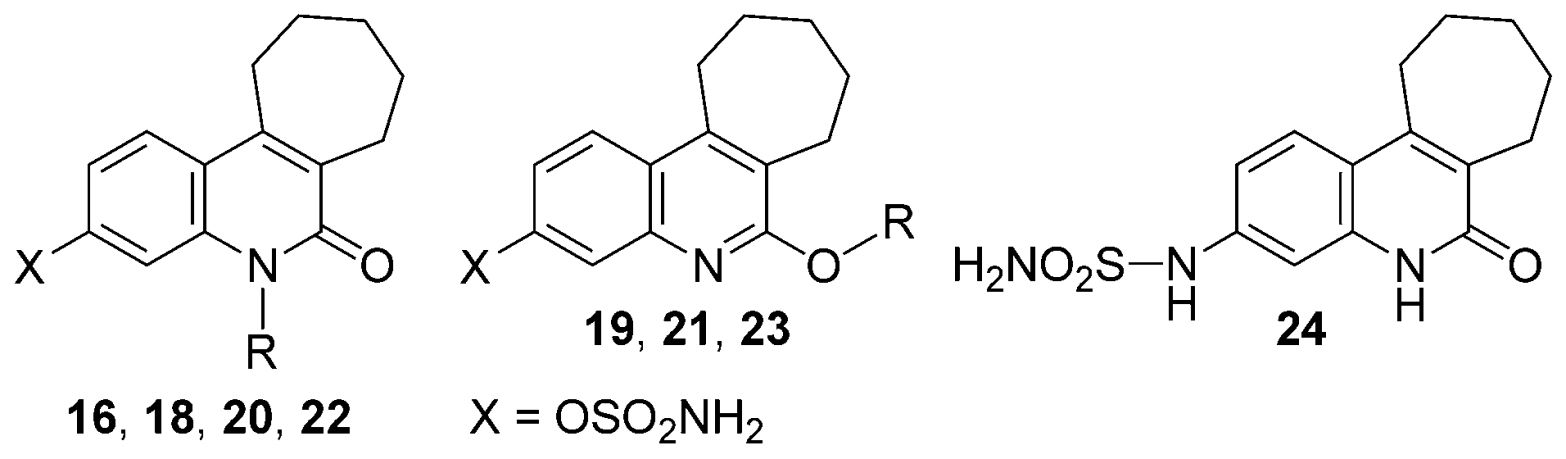			
Compd	*n*	PM IC_50_ [nm][a]	JEG-3 IC_50_ [nm]
**2**	3	200	32
**3**	4	70	7
**1**	5	8	1.5
**4**	6	30	0.9
**6**	7	2.4	0.022
**7**	8	1	0.025
**8**	9	13	0.015
**9**	10	60	100
**10**	11	75	220
**11**	13	370	1600
**12**	NA	ND	>10 000
**13**	NA	ND	78±13
**14**	NA	ND	283±66

[a] Data from Ref. [23]. Unless stated otherwise, errors are <5 % of the reported value (from triplicate experiments); NA: not applicable; ND: not determined.

We recently replaced the placental microsome preparation with a JEG-3 cell preparation as the standard assay for screening the in vitro STS inhibitory activities of compounds. The advantage of using intact growing JEG-3 cells is that they allow testing of the compounds under conditions that closely resemble the tissue/physiological situation in which the drug must first cross the plasma membrane before it can reach the target (STS) enzyme. These human choriocarcinoma cells have abundant STS enzyme activity, are easy to grow, and are less expensive to use than purified enzyme or placental microsomes. We therefore re-tested the STS inhibitory activities of the tricyclic coumarin sulfamates in JEG-3 cells, and their IC_50_ values are listed in Table 1. As expected for a cell-based assay, the IC_50_ values against STS obtained for the series of compounds are much lower than those obtained from the cell-free placental microsome assay. However, the overall in vitro inhibitory profile observed is similar, with potency increasing as the size of the third aliphatic ring increases from 5 to 11 members, but then decreasing as the ring size increases further. The most potent compounds observed are **6**–**8**, the IC_50_ values of which are between 0.015 and 0.025 nm, whereas **11** is the weakest STS inhibitor in vitro. These results suggest that the ability of compounds to cross the cell membrane and then to interact with the active site of STS is optimal with compounds **6**–**8**, when the aliphatic ring contains 8–10 carbon atoms. Unexpectedly, there is a dramatic decrease in potency observed when the size of the third ring increases from 11 to 12 members. There is a five orders of magnitude difference between the IC_50_ values of **8** and **9**.

**Figure 3:**
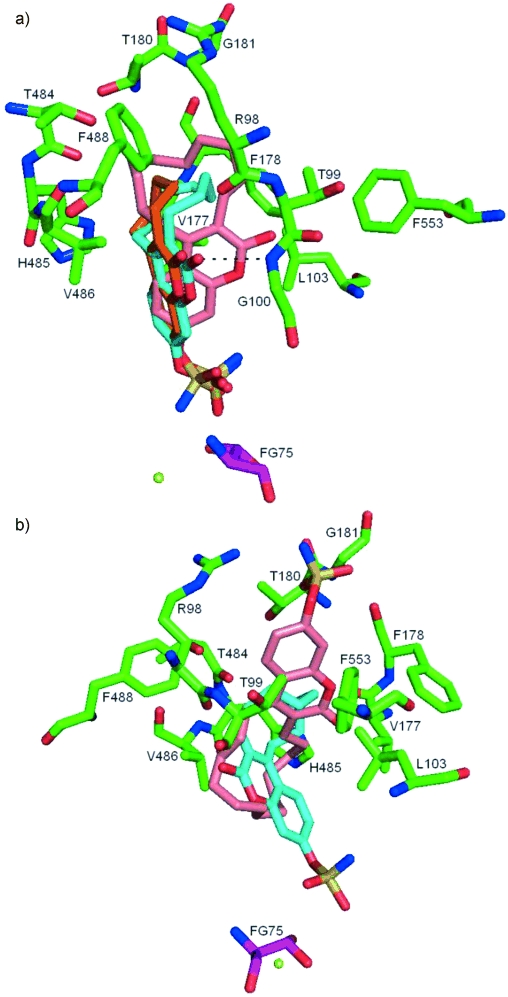
The docking of a) **1** (orange), **7** (cyan), and **9** (pink); and b) **7** (cyan) and **11** (pink) into the crystal structure of human STS. The Ca^2+^ ion is depicted as a yellow sphere, and FG75 is the *gem*-diol form of FG75. Dotted line: potential hydrogen bond.

To examine the possible interactions of tricyclic coumarin derivatives with amino acid residues within the active site of STS, these molecules were docked into the crystal structure of STS (PDB ID: 1P49).[24] Importantly, the poses discussed are assumed to be those that form immediately prior to the irreversible inactivation of the enzyme by sulfamoyl transfer. Although it is currently not known what residue is involved, these docking results would be predictive of inactivation of the *gem*-diol form of the formylglycine residue 75 (FG75) by sulfamoyl transfer. The docking results for **1**, **7**, and **9** are shown in Figure 3 a and those for **7** and **11** in Figure 3 b. In common with **1** and **7**, as shown in Figure 3 a, the rest of the compounds in the series, apart from compound **11**, bind with the sulfamate down by the catalytically crucial FG75 residue and the calcium ion. This leaves the third aliphatic ring residing in a predominantly hydrophobic pocket formed by R98, T99, L103, V177, F178, T180, G181, T484, H485, V486, F488, and F553. As the size of the third ring increases from 5 to 11 members (compounds **1**–**4** and **6**–**8**), it gives a more favourable contact with these residues, with the first and second rings (the coumarin moiety) and the sulfamate occupying nearly identical positions. This may partly explain the increase in potency of these compounds in general as the third aliphatic ring increases in size. As shown in Figure 3 a, and exemplified by compounds **1** and **7**, the carbonyl groups of these compounds are within hydrogen bonding distance from the backbone NH group of G100 (∼3 Å). This additional interaction may be a contributing factor that further assists the binding of these molecules to the enzyme active site. The docking pose of compound **9** (12-membered third ring) is different from that of its lower congeners. Presumably due to steric hindrance rendered by the bulk of its third ring, **9** binds with the coumarin ring rotated in the binding site (Figure 3 a). As a result, its carbonyl group is no longer positioned to form a hydrogen bond to G100. The same observations can be made for compound **10** (13-membered third ring), as it shows a docking pose similar to that of compound **9** (not shown). With compound **11**, the 15-membered third ring is too large to fit in the binding site in the same orientation as it does for compounds **1**–**4** and **6**–**10**. In contrast to its congeners, **11** binds upside down in the binding site (Figure 3 b) which is a much poorer binding pose. The GOLD docking scores for compounds **1**–**4** and **6**–**10** are all in the range of 52–57 which are not sufficiently different to allow any correlation to be made between their docking poses and IC_50_ values. However, **11** has a significantly lower GOLD docking score of 38 which may reflect the much poorer IC_50_ observed for this compound.

The N,N-dimethylation of **1** to give compound **12** renders the compound inactive in vitro as an STS inhibitor (Table 1). This supports previous findings that a free sulfamate group is a prerequisite for potent irreversible inhibition of STS in vitro. Hence, *N*-(piperidino),[25] *N*,*N*-(dibenzyl)sulfamate,[25] and *N*,*N*-dimethyl derivatives of oestrone 3-*O*-sulfamate (EMATE)[26] were found to be weak reversible or inactive inhibitors of STS in placental microsomes. Only N-acetylated EMATE, but not the benzoyl derivative, inhibits STS irreversibly, albeit much less potently than EMATE.[25] However, compound **12** was found to behave differently in vivo. When administered orally to nude mice, **12** inhibits liver STS activity potently at doses of 1 and 10 mg kg^−1^.[27] Moreover, if **12** is applied topically at 1 and 10 mg kg^−1^, it also inhibits skin as well as liver STS effectively.[27] This shows that **12** is able to be absorbed via the percutaneous route and could then inhibit STS in the liver and possibly in other tissues throughout the body. We reason that demethylation of **12** occurs enzymatically in vivo, releasing **1** which is then the agent that inhibits STS.

Keeping a free sulfamate group at the 3-position of 1 but introducing a methoxy group at the 2-position renders the resulting compound **13** a weaker STS inhibitor in JEG-3 cells (IC_50_=78 nm for **13** versus 1.5 nm for **1**, Table 1). A similar pattern was observed with 2-methoxyestrone 3-*O*-sulfamate (IC_50_=30 nm), which was found to be a weaker STS inhibitor than EMATE (IC_50_=4 nm) in a preparation of placental microsomes.[28] Having a bulkier aliphatic substituent positioned next to an aryl sulfamate has also been found to confer weaker inhibition of STS, presumably due to steric hindrance.[28]

The relocation of the sulfamate group in **1** from the 3- to the 2-position renders a significant decrease in STS inhibitory activity of the resulting compound **14** (Table 1). It is reasoned that the high inhibitory activity observed for **1** is due to its sulfamate group being in a position conjugated to the α,β-unsaturated lactone moiety of the coumarin ring. As a result, the parent phenol **1 a** has a lower p*K*_a_ value and is hence a better leaving group than unsubstituted phenol. We postulate that this effect would more effectively facilitate the transfer of the sulfamoyl group of **1** to an essential amino acid residue in the STS active site and inactivate the enzyme as a result. Relocation of the sulfamate group from the 3- to the 2-position to give **14** would essentially disrupt this process, as the p*K*_a_ of the parent phenol **14 a** is expected to be close to that of unsubstituted phenol. It is also possible that a sulfamate group placed at the 2-position might not be presented properly and effectively to essential amino acid residue(s) in the enzyme catalytic site responsible for its subsequent activation, resulting in less effective inactivation of the enzyme.

**Figure 4:**
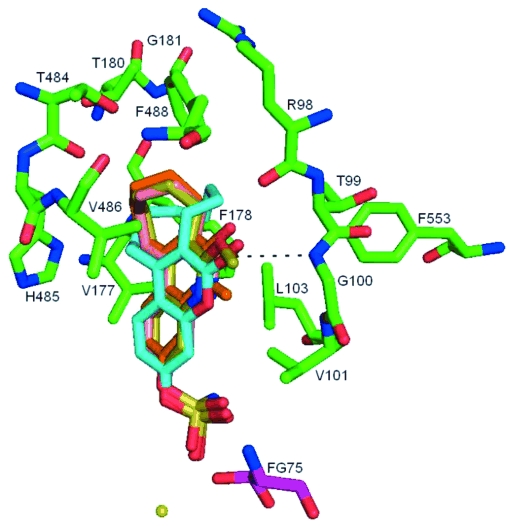
The docking of **7** (cyan), **16** (pink), **18** (orange), and **19** (yellow) into the crystal structure of human STS. The Ca^2+^ ion is depicted as a yellow sphere, and FG75 is the *gem*-diol form of FG75. Dotted line: potential hydrogen bond.

**Figure 5:**
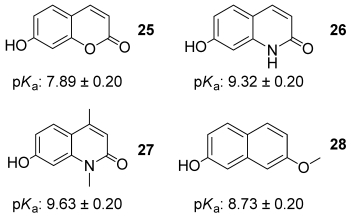
Calculated p*K*_a_ values of various bicyclic phenols **25**–**28**, which represent the parent phenols of **1**, **16**, **18**, and **19**, respectively. The calculation was performed by ACD/Labs software version 11.01.

The coumarin moiety has been the core bicyclic template for the development of nonsteroidal STS inhibitors by our research group. Other phenols of bicyclic nonsteroidal moieties such as tetrahydronaphthalene;[26] flavones, isoflavones, flavanones;[29, 30] and chromenone and thiochromenone[31] have also been sulfamoylated and explored by us and other research groups for designing STS inhibitors with varying degrees of success. In this work, we studied the effects of replacing the coumarin ring system of **1** with either a quinolin-2(1*H*)-one or a quinoline moiety. Their respective N-alkylated and alkoxyl derivatives were also investigated for STS inhibitory activity. As shown in Table 2, all compounds inhibit STS weakly in JEG-3 cells. The best STS inhibitor is the unsubstituted quinolinone derivative **16** (IC_50_=240 nm or 98 % inhibition at 10 μm), although it is 160-fold less potent than **1** (IC_50_=1.5 nm, Table 1). This is closely followed by the quinoline derivative **19**, which inhibits STS by 68 % at 10 μm, although the inhibition remains weak. These results further confirm that the coumarin ring is essential for the potent STS inhibitory activity observed for **1**. This is attributed to several factors. With **16**, **18**, and **19** docked into the STS active site in a fashion similar to that of **7** (Figure 4), we postulate that electronic factors such as the p*K*_a_ values of parent phenols could play a significant role for the results observed. To explore this possible causative factor further, the p*K*_a_ values of 7-hydroxy-2*H*-chromen-2-one (**25**, represents **1 a**, the parent phenol of **1**), 7-hydroxyquinolin-2(1*H*)-one (**26**, represents **16 a**, the parent phenol of **16**), 7-hydroxy-1,4-dimethylquinolin-2(1*H*)-one (**27**, represents **18 b**, the parent phenol of **18**), and 7-methoxynaphthalen-2-ol (**28**, represents **19 c**, the parent phenol of **19**) as calculated by ACD/Labs software version 11.01 were compared (Figure 5). As shown, the p*K*_a_ value of **1 a** is expected to be between 1 and 2 log units lower than those of **16 a**, **18 b**, and **19 c**. This factor suggests that **1 a** is a much better leaving group than **16 a**, **18 b**, and **19 c**, rendering the sulfamate group of **1 a** a much stronger sulfamoylating species for the inactivation of the enzyme, and hence **1** is a more potent STS inhibitor than the quinolinone and quinoline derivatives.

**Table 2:** Inhibition of STS activity in JEG-3 cells by tricyclic quinolinone sulfamates **16**, **18**, **20**, and **22**, tricyclic quinoline sulfamates **19**, **21**, and **23**, and the tricyclic quinolinone sulfamide **24**

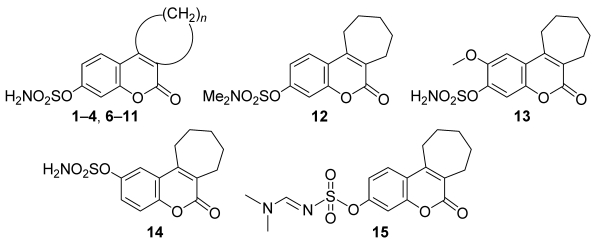			
Compd	R	Inhibition [%][a,b]	IC_50_ [nm][b]
**16**	H	96	240±40
**18**	Me	ND	2400
**19**	Me	68	ND
**20**	(CH_2_)_4_CH_3_	<10	ND
**21**	(CH_2_)_4_CH_3_	<10	ND
**22**	(CH_2_)_3_Ph	20	ND
**23**	(CH_2_)_3_Ph	<10	ND
**24**	ND	ND	>10^3^

[a] Determined at 10 μm.

[b] Unless stated otherwise, errors are <5 % of the reported value (from triplicate experiments); ND: not determined.

N-Methylation of **16** to give **18** (IC_50_=2400 nm, Table 2) is detrimental to activity, as this substitution produces a 100-fold decrease in the IC_50_ value observed for **18** against STS. For both quinolinone and quinoline series, further enlargement of the substituent from a methyl group to either an *n*-pentyl or a phenethyl group significantly abolishes the STS inhibitory activities of the resulting compounds. It is possible that these substituted molecules no longer bind effectively to the active site of STS due to steric hindrance caused by the bulk of the substituent.

Finally, replacement of the bridging oxygen atom of the sulfamate group in **16** with an NH moiety to give a sulfamido group abolishes the activity of the resulting compound **24** as an STS inhibitor. A similar finding was observed with oestrone 3-sulfamide.[19] We postulate that, unlike the sulfamate group of **16**, an enzyme-catalysed breaking of the S–N bond of the sulfamido group of **24** is unlikely to take place because, among other things, the parent amine **24 a** is a very poor leaving group. As a result, it is not anticipated that **24** would be able to inactivate STS to any degree by sulfamoylating the active site, but such an approach could provide leads for reversible STS inhibitors.

## Conclusions

The nonsteroidal inhibitor Irosustat, STX64 (**1**) is the first agent to enter clinical trials for postmenopausal patients with advanced hormone-dependent breast cancer, and has shown encouraging results. In this work, we conducted a range of SAR studies on this drug. Expansion of the size of the aliphatic ring of **1** generally provides more potent derivatives against STS in JEG-3 cells, with best activities observed if the ring is between 9 and 11 members. However, further increasing the ring size is unfavourable, as inhibitory activities were observed to drop significantly. Molecular docking studies suggest that the aliphatic ring of **1** and its derivatives sit in a hydrophobic pocket within the enzyme active site with better contacts made with the enclosing amino acid residues as the ring size increases up to 11 members. Larger derivatives **9** and **10**, and in particular **11**, dock less well into the active site. Positioning of the sulfamate moiety close to the catalytic FG75 may be predictive of sulfamoyl transfer to this residue in the inactivation process. N,N-Dimethylation of the sulfamate group of **1** is detrimental to in vitro activity, as compound **12** is inactive. This supports previous findings which showed that a free sulfamate group (H_2_NSO_2_O^−^) is a prerequisite for potent and irreversible STS inhibition. Introducing a methoxy group at the 2-position of **1** significantly decreases the activity of the resulting **13**, probably as a result of steric factors. A detrimental effect to activity is also observed with relocation of the sulfamate group of **1** from the 3- to the 2-position of the molecule. We postulate that the decrease in activity of compound **14** is due to its sulfamate group not being in a conjugated position to the α,β-unsaturated lactone moiety of the coumarin ring, which affects the ability of **14** to sulfamoylate and inactivate the enzyme. An azomethine adduct between **1** and the solvent DMF used in the sulfamoylation of **1 a** was isolated. Its crystal structure shows that the stereochemistry is *E* at the double bond of its (dimethylamino)methylene sulfamoyl group. Replacing the coumarin ring system of **1** to give a series of quinolin-2(1 *H*)-one and quinoline derivatives produces essentially weak inhibitors of STS. Only the lowest members of the series inhibit STS. This confirms the unique property of the coumarin system in the design of nonsteroidal STS inhibitors that are structurally related to **1**.

In summary, most of the modifications made to the clinical drug **1** decrease potency in vitro. Only a moderate enlargement of its aliphatic ring results in derivatives that are more potent STS inhibitors in vitro. However, it remains to be explored whether such compounds would show significant advantages over **1** if put through pre-clinical trial development.

## Experimental Section

**In vitro sulfatase assay:** Biological assays were performed essentially as described previously.[32] The extent of in vitro inhibition of STS activities was assessed by using intact monolayers of JEG-3 human choriocarcinoma cells. STS activity was measured with [6,7-^3^H]E1S (50 Ci mmol^−1^, PerkinElmer Life Sciences) over a 1 h period.

**Molecular modelling:** All ligands were built and minimised using Schrödinger software running under Maestro version 9.0. The crystal structure of human placental oestrone/DHEA sulfatase (PDB ID: 1P49)[24] was used for building the *gem*-diol form of STS. This involved a point mutation of the ALS75 residue in the crystal structure to the *gem*-diol form of the structure using editing tools within the Schrödinger software. The resulting structure was then minimised with the backbone atoms fixed to allow the *gem*-diol and surrounding side chain atoms to adopt low-energy confirmations. GOLD was used to dock the ligands 25 times each into the rigid protein, with the binding site being defined as a 10 Å sphere around the ALS75 sulfate. The docked poses were scored using the GOLDScore fitness function.

**General methods for synthesis**: All chemicals were purchased from either Aldrich Chemical Co. (Gillingham, UK) or Alfa Aesar (Heysham, UK). All organic solvents of analytical reagent grade were supplied by Fisher Scientific (Loughborough, UK). Anhydrous *N*,*N*-dimethylformamide (DMF), *N*,*N*-dimethylacetamide (DMA), and tetrahydrofuran (THF) were purchased from Aldrich. Sulfamoyl chloride was prepared by an adaptation of the method of Appel and Berger[33] and was stored as a solution under N_2_ in toluene as described by Woo et al.[19]

Thin-layer chromatography (TLC) was performed on pre-coated plates (Merck TLC aluminium sheets silica gel 60 F_254_, Art. No. 5554). Product(s) and starting material were detected by viewing under UV light and/or treating with a methanolic solution of phosphomolybdic acid followed by heating. Flash column chromatography was performed using gradient elution (solvents indicated in the text) on wet-packed silica gel (Sorbsil C_60_). IR spectra were determined with a PerkinElmer 782 infrared spectrophotometer, and peak positions are expressed in cm^−1^. ^1^H and ^13^C NMR spectra were recorded with either a Jeol Delta 270 MHz or a Varian Mercury VX 400 MHz spectrometer. Chemical shifts (*δ*) are reported in parts per million (ppm) relative to tetramethylsilane (TMS) as an internal standard. Coupling constants (*J*) are recorded to the nearest 0.1 Hz. Mass spectra were recorded at the Mass Spectrometry Service Centre, University of Bath. FAB mass spectra were measured using *m*-nitrobenzyl alcohol as the matrix. Elemental analyses were performed by the Microanalysis Service, University of Bath. Melting points were determined using a Reichert–Jung Thermo Galen Kofler block and are uncorrected. HPLC was undertaken using a Waters 717 instrument equipped with an autosampler and PDA detector. The column used, conditions of elution, and purity of sample are as indicated for each compound analysed.

**Crystallographic data:** CCDC 826524 (**1**) and 826525 (**15**) contain the supplementary crystallographic data for this paper. These data can be obtained free of charge from The Cambridge Crystallographic Data Centre via http://www.ccdc.cam.ac.uk/data_request/cif

**Ethyl 2-oxocyclononanecarboxylate (6 a).** A solution of cyclononanone (3.0 g, 21 mmol) in diethyl carbonate (20 mL) was added dropwise to a suspension of NaH (60 % dispersion in mineral oil, 1.71 g, 42.8 mmol) and diethyl carbonate (80 mL) under N_2_ over a period of 30 min. When the evolution of H_2_ had ceased (∼15 h), aqueous HCl (1 m, 100 mL) was added in portions, and the resulting mixture was extracted with Et_2_O (3×100 mL). The combined ethereal extracts were dried (MgSO_4_) and evaporated to give a yellow oily residue, which was purified by distillation under reduced pressure to give **6 a** as a clear oil (4.15 g, 91 %): *R*_f_=0.72 (CHCl_3_); bp_3_: 146–150 °C; (Lit. [34] bp_2_: 108–110 °C); ^1^H NMR (400 MHz, CDCl_3_): *δ*=1.23 (t, *J*=7.2 Hz, 1.8 H, keto CH_2_C*H*_3_), 1.30 (t, *J*=7.2 Hz, 1.2 H, enol CH_2_C*H*_3_), 1.37–2.66 (m, 14 H), 3.62 (m, 0.6 H, keto CHC=O), 4.14 (q, *J*=7.2 Hz, 1.2 H, keto C*H*_2_CH_3_), 4.21 (q, *J*=7.2 Hz, 0.8 H, enol C*H*_2_CH_3_), and 12.76 ppm (s, 0.4 H, ex. with D_2_O, enol OH); MS (FAB^+^): *m*/*z* (%): 213.0 (100) [*M*+H]^+^; HRMS-FAB^+^: *m*/*z* [*M*+H]^+^ calcd for C_12_H_21_O_3_: 213.1491, found: 213.1499.

**3-Hydroxy-8,9,10,11,12,13-hexahydrocyclonona[*c*]chromen-6(7*H*)-one (6 b).** Resorcinol (1.56 g, 14.1 mmol) was first dissolved in hot **6 a** (3.0 g, 14 mmol). Upon cooling to room temperature, the resulting syrup at 0 °C was treated dropwise with a mixture of CF_3_COOH (2.2 mL, 28 mmol) and concd H_2_SO_4_ (1.5 mL, 28 mmol) while keeping the reaction temperature <10 °C. After stirring for 3 h at room temperature, the orange gluey mass was cautiously quenched with ice-water. The orange precipitate that formed was collected by suction filtration, washed exhaustively with water and air dried. A solution of the precipitate in a minimal volume of acetone was fractionated by flash chromatography (CHCl_3_/acetone, 8:1 → 4:1 gradient). The main fraction collected gave a white solid which was recrystallised from THF/hexane to give **6 b** as white fine crystals (909 mg, 25 %): *R*_f_=0.82 (CHCl_3_/acetone, 3:1); mp: 197–200 °C; ^1^H NMR (400 MHz, CDCl_3_): *δ*=1.29–2.51 (m, 10 H, 5×CH_2_), 2.66 (t, *J*=5.8 Hz, 2 H, C7-CH_2_), 2.93 (t, *J*=6.1 Hz, 2 H, C13-CH_2_), 6.69 (d, *J*=2.4 Hz, 1 H, C4-H), 6.78 (dd, *J*=2.4 and 8.8 Hz, 1 H, C2-H), 7.59 (d, *J*=8.8 Hz, 1 H, C1-H) and 10.39 ppm (s, 1 H, OH); MS (FAB^+^): *m*/*z* (%): 259.1 (100) [*M*+H]^+^; MS (FAB^−^): *m*/*z* (%): 257.1 (100), [*M*-H]^−^; HRMS-FAB^+^: *m*/*z* [*M*+H]^+^ calcd for C_16_H_19_O_3_: 259.1334, found: 259.1323; Anal. calcd for C_16_H_18_O_3_: C 74.40, H 7.02, found: C 74.10, H 6.91; HPLC: Waters Radialpak column (RP_18_, 8×100 mm), MeOH/H_2_O (70:30), flow rate=2 mL min^−1^, *λ*_max_=323.2 nm, *t*_R_=6.5 min, purity >98 %.

**6-Oxo-6,7,8,9,10,11,12,13-octahydrocyclonona[*c*]chromen-3-yl sulfamate (6).** NaH (60 % dispersion in mineral oil, 1 equiv) was added to a solution of **6 b** (400 mg, 1.55 mmol) in anhydrous DMF (20 mL) at 0 °C under N_2_. When the evolution of H_2_ had ceased, sulfamoyl chloride (∼0.69 m in toluene,[19] ∼3–5 equiv, evaporated down to ∼1 mL prior to addition) was introduced in one portion. After stirring at room temperature under N_2_ overnight, the reaction mixture was quenched with ice-water. Upon addition of EtOAc (∼100 mL), the organic fractions were washed with brine (4×100 mL). After drying (MgSO_4_), filtering and evaporating the washed organic layer, a crude white solid was obtained which was purified by flash chromatography (CHCl_3_/EtOAc, 8:1 → 2:1 gradient). The main fraction isolated gave a white solid which was recrystallised from THF/hexane to give **6** as fine white crystals (201 mg, 38 %): *R*_f_=0.46 (CHCl_3_/EtOAc, 4:1); mp: 167–168 °C; ^1^H NMR (400 MHz, CDCl_3_): *δ*=0.84–1.74 (m, 10 H, 5×CH_2_), 1.52 (t, *J*=5.8 Hz, 2 H, C7-CH_2_), 1.57 (t, *J*=6.1 Hz, 2 H, C13-CH_2_), 7.26 (dd, *J*=2.4 and 8.8 Hz, 1 H, C2-H), 7.31 (d, *J*=2.1 Hz, 1 H, C4-H), 7.89 (d, *J*=8.8 Hz, 1 H, C1-H) and 8.20 ppm (s, 2 H, NH_2_); MS (FAB^+^): *m*/*z* (%): 338.0 (100) [*M*+H]^+^; MS (FAB^−^): *m*/*z* (%): 336.1 (100) [*M*−H]^−^, 257.1 (30) [*M*−H_2_NSO_2_]^−^; HRMS-FAB^+^: *m*/*z* [*M*+H]^+^ calcd for C_16_H_20_NO_5_S: 338.1062, found: 338.1061; Anal. calcd for C_16_H_19_NO_5_S: C 56.96, H 5.68, N 4.15, found: C 56.85, H 5.58, N 4.00; HPLC: Waters Radialpak column (RP_18_, 8×100 mm), MeOH/H_2_O (70:30), flow rate=2 mL min^−1^, *λ*_max_=284 and 312.5 nm, *t*_R_=3.1 min, purity >98 %.

**Ethyl 2-oxocyclodecanecarboxylate (7 a).** Prepared in a similar manner to **6 a** using NaH (1.3 g, 32 mmol), diethyl carbonate (60 mL), and cyclodecanone (2.5 g, 16 mmol). The crude pale-yellow oily residue was purified by distillation under reduced pressure to give **7 a** as a colourless oil (2.81 g, 76 %): *R*_f_=0.81 (CHCl_3_); bp_0.23_: 84–87 °C (Lit. [34] bp_1_: 118–120 °C); ^1^H NMR (400 MHz, CDCl_3_): *δ*=1.24 (t, *J*=7.0 Hz, 1.2 H, keto CH_2_C*H*_3_), 1.31 (t, *J*=7.0 Hz, 1.8 H, enol CH_2_C*H*_3_), 1.34–2.76 (m, 16 H), 3.82–3.85 (m, 0.7 H, keto CHC=O), 4.13 (q, *J*=7.0 Hz, 0.5 H, keto C*H*_2_CH_3_), 4.22 (q, *J*=7.0 Hz, 1.5 H, enol C*H*_2_CH_3_) and 12.98 ppm (s, 0.3 H, ex. with D_2_O, enol OH); MS (FAB^+^): *m*/*z* (%): 227.0 (100) [*M*+H]^+^; HRMS-FAB^+^: *m*/*z* [*M*+H]^+^ calcd for C_13_H_23_O_3_: 227.1647, found: 227.1644.

**3-Hydroxy-7,8,9,10,11,12,13,14-octahydro-6*H*-cyclodeca[*c*]chromen-6-one (7 b).** Prepared in a similar manner to **6 b** using resorcinol (970 mg, 8.84 mmol), **7 a** (2.0 g, 8.8 mmol), and a mixture of CF_3_COOH (1.5 mL, 18 mmol) and concd H_2_SO_4_ (1.0 mL, 18 mmol). The crude dark-orange solid was purified by flash chromatography (CHCl_3_/acetone, 8:1 → 4:1 gradient), and the white solid that was isolated was recrystallised from THF/hexane to give **7 b** as white crystals (789 mg, 33 %): *R*_f_:=0.72 (CHCl_3_/acetone, 3:1); mp: 240–241 °C; ^1^H NMR (400 MHz, [D_6_]DMSO): *δ*=0.88–2.18 (m, 12 H, 6×CH_2_), 2.83 (t, *J*=6.7 Hz, 2 H, C7-CH_2_), 3.02 (t, *J*=6.7 Hz, 2 H, C14-CH_2_), 5.96 (s, 1 H, OH), 6.78 (dd, *J*=2.7 and 8.5 Hz, 1 H, C2-H), 6.83 (d, *J*=2.7 Hz, 1 H, C4-H) and 7.53 ppm (d, *J*=8.5 Hz, 1 H, C1-H); MS (FAB^+^) *m*/*z* (%): 273.1 (100) [*M*+H]^+^; MS (FAB^−^): *m*/*z* (%): 271.1 (100) [*M*−H]^−^; HRMS-FAB^+^: *m*/*z* [*M*+H]^+^ calcd for C_17_H_21_O_3_: 273.1491, found: 273.1488; Anal. calcd for C_17_H_20_O_3_: C 74.94, H 7.40, found: C 74.30, H 7.43; HPLC: Waters Radialpak column, MeOH/H_2_O (80:20), flow rate=2 mL min^−1^, *λ*_max_=322 nm, *t*_R_=4.5 min, purity >95 %.

**6-Oxo-7,8,9,10,11,12,13,14-octahydro-6*H*-cyclodeca[*c*]chromen-3-yl sulfamate (7).** Compound **7 b** (400 mg, 1.47 mmol) was sulfamoylated in a similar manner to **6 b** and the crude white solid obtained was purified by flash chromatography (CHCl_3_/EtOAc, 8:1 → 2:1 gradient). The white solid that was isolated was recrystallised from THF/hexane to give **7** as fine white crystals (235 mg, 46 %): *R*_f_=0.71 (CHCl_3_/EtOAc, 4:1); mp: 183–185 °C; ^1^H NMR (400 MHz, [D_6_]DMSO): *δ*=1.17–3.0 (m, 14 H, 7×CH_2_), 3.09 (t, *J*=6.4 Hz, 2 H, C14-CH_2_), 7.26 (m, 1 H, C2-CH), 7.46 (d, *J*=1.2 Hz, 1 H, C4-H), 7.93 (d, *J*=8.8 Hz, 1 H, C1-H) and 8.21 ppm (s, 2 H, NH_2_); MS (FAB^+^): *m*/*z* (%): 352.0 (100) [*M*+H]^+^; MS (FAB^−^): *m*/*z* (%): 350.1 (100) [*M*−H]^−^, 271.1 (100) [*M*−H_2_NSO_2_]^−^; HRMS-FAB^+^: *m*/*z* [*M*+H]^+^ calcd for C_17_H_22_NO_5_S: 352.1219, found: 352.1223; Anal. calcd for C_17_H_21_NO_5_S: C 58.10, H 6.02, N 3.99 %, found: C 58.40, H 6.28, N, 2.63; HPLC: Waters Radialpak column, MeOH/H_2_O (70:30), flow rate=2 mL min^−1^, *λ*_max_=284 and 312.5 nm, *t*_R_=6.3 min, purity >98 %.

**Ethyl 2-oxocycloundecanecarboxylate (8 a).** Prepared in a similar manner to **6 a** using NaH (1.19 g, 29.7 mmol), diethyl carbonate (70 mL), and cycloundecanone (2.5 g, 15 mmol). The crude yellow oily residue was purified by distillation under reduced pressure to give **6 a** as a pale-yellow oil (2.07 g, 58 %): *R*_f_=0.31 (CH_2_Cl_2_); bp_0.15_: 103–108 °C (Lit. [34] bp_5_: 140–143 °C); ^1^H NMR (400 MHz, CDCl_3_) *δ*=1.20 (t, *J*=7.0 Hz, 3 H, CH_2_C*H*_3_), 1.26–2.76 (m, 19 H) and 4.10 ppm (q, *J*=7.3 Hz, 2 H, CH_3_C*H*_2_); MS (FAB^+^): *m*/*z* (%): 241.1 (100) [*M*+H]^+^; MS (FAB^−^): *m*/*z* (%): 239.0 (100) [*M*−H]^−^; HRMS-FAB^+^: *m*/*z* [*M*+H]^+^ calcd for C_14_H_25_O_3_: 241.1804, found: 241.1806.

**3-Hydroxy-8,9,10,11,12,13,14,15-octahydrocycloundeca[*c*]chromen-6(7*H*)-one (8 b).** Prepared in a similar manner to **6 b** using resorcinol (917 mg, 8.33 mmol), **8 a** (2.0 g, 8.3 mmol) and a mixture of CF_3_COOH (2.0 mL, 17 mmol) and concd H_2_SO_4_ (1.6 mL, 17 mmol). The crude yellow solid was purified by flash chromatography (CHCl_3_/acetone, 8:1 → 4:1 gradient) and the yellow solid that was isolated was recrystallised from THF/hexane to give **8 b** as fine pale-yellow crystals (344 mg, 14 %): *R*_f_=0.76 (CHCl_3_/acetone, 3:1); mp: 214–215 °C; ^1^H NMR (400 MHz, [D_6_]DMSO): *δ*=1.26–1.68 (m, 14 H, 7×CH_2_), 2.56 (t, *J*=7.0 Hz, 2 H, C7-CH_2_), 2.84 (t, *J*=7.0 Hz, 2 H, C15-CH_2_), 6.67 (d, *J*=2.1 Hz, 1 H, C4-H), 6.78 (dd, *J*=2.1 and 8.7 Hz, 1 H, C2-H), 7.63 (d, *J*=8.8 Hz, 1 H, C1-H) and 10.42 ppm (s, 1 H, OH); MS (FAB^+^): *m*/*z* (%) 287.1 (100) [*M*+H]^+^; MS (FAB^−^): *m*/*z* (%): 285.1 (100) [*M*−H]^−^; HRMS-FAB^+^: *m*/*z* [*M*+H]^+^ calcd for C_18_H_23_O_3_: 287.1647, found: 287.1644; Anal. calcd for C_18_H_22_O_3_: C 75.50, H 7.74, found: C 75.50, H 7.75; HPLC: Waters Radialpak column, MeOH/H_2_O (80:20), flow rate=2 mL min^−1^, *λ*_max_=323.2 nm, *t*_R_=6.5 min, purity >98 %.

**6-Oxo-6,7,8,9,10,11,12,13,14,15-decahydrocycloundeca[*c*]chromen-3-yl sulfamate (8).** Compound **8 b** (300 mg, 1.05 mmol) was sulfamoylated in a similar manner to **6 b** and the crude white solid obtained was purified by flash chromatography (CHCl_3_/EtOAc, 8:1 → 2:1 gradient). The white solid that was isolated was recrystallised from THF/hexane to give **8** as fine white crystals (133 mg, 35 %): *R*_f_=0.37 (CHCl_3_/EtOAc, 4:1); mp: 145–148 °C; ^1^H NMR (400 MHz, [D_6_]DMSO): *δ*=1.28–1.76 (m, 14 H, 7×CH_2_), 2.64 (t, *J*=7.0 Hz, 2 H, C7-CH_2_), 2.93 (t, *J*=7.0 Hz, 2 H, C15-CH_2_), 7.26 (dd, *J*=2.1 and 8.8 Hz, 1 H, C2-H), 7.29 (d, *J*=2.1 Hz, 1 H, C4-H), 7.93 (d, *J*=8.8 Hz, 1 H, C1-H) and 8.20 ppm (s, 2 H, NH_2_); MS (FAB^+^): *m*/*z* (%) 731.2 (10) [2 *M*+H]^+^, 366.0 (100) [*M*+H]^+^; MS (FAB^−^): *m*/*z* (%): 364.1 (100) [*M*−H]^−^, 285.2 (40) [*M*−H_2_NSO_2_]^−^; HRMS-FAB^+^: *m*/*z* [*M*+H]^+^ calcd for C_18_H_24_NO_5_S: 366.1375, found: 366.1368; Anal. calcd for C_18_H_23_NO_5_S: C 59.16, H 6.34, found: C 59.20, H 6.57; HPLC: Waters Radialpak column, MeOH/H_2_O (80:20), flow rate=2 mL min^−1^, *λ*_max_=285.2 and 312.5 nm, *t*_R_=3.8 min, purity >98 %.

**Ethyl 2-oxocyclododecanecarboxylate (9 a).** Prepared in a similar manner to **6 a** using NaH (2.19 g, 54.9 mmol), diethyl carbonate (100 mL) and cyclododecanone (5.0 g, 27 mmol). The crude dark-yellow oily residue was purified by distillation under reduced pressure to give **9 a** as a pale-yellow oil (5.62 g, 81 %): *R*_f_=0.72 (CH_2_Cl_2_); bp_0.23_: 128–132 °C (Lit. [34] bp_3_: 155–157 °C); ^1^H NMR (400 MHz, CDCl_3_): *δ*=1.25 (t, *J*=7.0 Hz, 3 H, CH_2_C*H*_3_), 1.29–2.73 (m, 21 H) and 4.16 ppm (q, *J*=6.4 Hz, 2 H, CH_3_C*H*_2_); MS (FAB^+^): *m*/*z* (%): 255.1 (100) [*M*+H]^+^; MS (FAB^−^): *m*/*z* (%): 253.2 (100) [*M*−H)^−^]; HRMS-FAB^+^: *m*/*z* [*M*+H]^+^ calcd for C_15_H_27_O_3_: 255.1960, found: 255.1968.

**3-Hydroxy-7,8,9,10,11,12,13,14,15,16-decahydro-6*H*-cyclododeca[*c*]chromen-6-one (9 b).** Prepared in a similar manner to **6 b** using resorcinol (1.08 g, 9.8 mmol), **9 a** (2.5 g, 9.8 mmol) and a mixture of CF_3_COOH (1.5 mL, 20 mmol) and concd H_2_SO_4_ (1.0 mL, 20 mmol). The crude pale-yellow solid was purified by flash chromatography (CHCl_3_/acetone, 8:1 → 4:1 gradient) and the pale-yellow solid that was isolated was recrystallised from THF/hexane to give **9 b** as white crystals (972 mg, 33 %): *R*_f_=0.76 (CHCl_3_/acetone, 3:1); mp: 249–251 °C; ^1^H NMR (400 MHz, [D_6_]DMSO): *δ*=1.39–2.89 (m, 16 H, 8×CH_2_), 2.93 (t, *J*=7.3 Hz, 2 H, C7-CH_2_), 3.22 (t, *J*=7.3 Hz, 2 H, C16-CH_2_), 6.66 (d, *J*=2.3 Hz, 1 H, C4-H), 6.78 (dd, *J*=2.3 and 8.9 Hz, 1 H, C2-H), 7.63 (d, *J*=8.9 Hz, 1 H, C1-H) and 10.77 ppm (s, 1 H, OH); MS (FAB^+^): *m*/*z* (%): 301.1 (100) [*M*+H]^+^; MS (FAB^−^): *m*/*z* (%): 299.1 (100) [*M*−H]^−^; HRMS-FAB^+^: *m*/*z* [*M*+H]^+^ calcd for C_19_H_25_O_3_: 301.1804, found: 301.1806; Anal. calcd for C_19_H_24_O_3_: C 75.97, H 8.05, found: C 75.90, H 8.03; HPLC: Waters Radialpak column, MeOH/H_2_O (90:10), flow rate=2 mL min^−1^, *λ*_max_=324.4 nm, *t*_R_=4.2 min, purity >98 %.

**6-Oxo-7,8,9,10,11,12,13,14,15,16-decahydro-6*H*-cyclododeca[*c*]chromen-3-yl sulfamate (9).** Compound **9 b** (400 mg, 1.33 mmol) was sulfamoylated in a similar manner to **6 b** and the crude white solid obtained was purified by flash chromatography (CHCl_3_/EtOAc, 8:1 → 2:1 gradient). The white solid that was isolated was recrystallised from THF/hexane to give **9** as fine white crystals (182 mg, 36 %): *R*_f_=0.47 (CHCl_3_/EtOAc, 4:1); mp: 173–175 °C; ^1^H NMR (400 MHz, [D_6_]DMSO): *δ*=1.41–2.51 (m, 16 H, 8×CH_2_), 2.62 (t, *J*=7.3 Hz, 2 H, C7-CH_2_), 3.68 (t, *J*=7.6 Hz, 2 H, C16-CH_2_), 7.26 (dd, *J*=2.4 and 8.5 Hz, 1 H, C2-H), 7.28 (d, *J*=2.4 Hz, 1 H, C4-H), 7.94 (d, *J*=8.5 Hz, 1 H, C1-H) and 8.20 ppm (s, 2 H, NH_2_); MS (FAB^+^): *m*/*z* (%): 380.1 (100) [*M*+H]^+^, 301.1 (15) [*M*+H−HNSO_2_]^+^; MS (FAB^−^): *m*/*z* (%): 378.1 (100) [*M*−H]^−^, 299.1 (50) [*M*−H_2_NSO_2_]^−^; HRMS-FAB^+^: *m*/*z* [*M*+H]^+^ calcd for C_19_H_26_NO_5_S: 380.1532, found: 380.1541; Anal. calcd for C_19_H_25_NO_5_S: C 60.14, H 6.64, N 3.69, found C 60.30, H 6.85, N 3.62; HPLC: Waters Radialpak column, MeOH/H_2_O (90:10), flow rate=2 mL min^−1^, *λ*_max_=285.2 and 312.5 nm, *t*_R_=5.3 min, purity >98 %.

**Ethyl 2-oxocyclotridecanecarboxylate (10 a).** Cyclic *β*-keto ester **10 a** was obtained commercially.

**3-Hydroxy-8,9,10,11,12,13,14,15,16,17-decahydrocyclotrideca[*c*]chromen-6(7*H*)-one (10 b).** Prepared in a similar manner to **6 b** using resorcinol (451 mg, 4.10 mmol), **10 a** (1.0 g, 3.73 mmol) and a mixture of CF_3_COOH (0.64 mL, 8.20 mmol) and concd H_2_SO_4_ (0.83 mL, 8.20 mmol). The light-beige residue (1.14 g) that was obtained was recrystallised from hot *i*PrOH to give **10 b** as soft yellow crystals (300 mg, 25.6 %): *R*_f_=0.51 (CHCl_3_/EtOAc, 4:1); mp: 234–238 °C; ^1^H NMR (400 MHz, CDCl_3_) *δ*=1.1–1.8 (18 H), 2.39 (m, 2 H, C7-CH_2_), 2.65 (m, 2 H, C17-CH_2_), 6.70 (d, *J*=2.4 Hz, 1 H, C4-H), 6.81 (dd, *J*=2.4 and 8.8 Hz, 1 H, C2-H), 7.61 (d, *J*=8.8 Hz, 1 H, C1-H) and 10.38 ppm (s, 1 H, ex. with D_2_O, OH); MS (FAB^+^): *m*/*z* (%): 315.3 (100) [*M*+H]^+^; MS (FAB^−^): *m*/*z* (%): 467.4 (35) [*M*−H+NBA]^−^, 313.4 (100) [*M*−H]^−^; HRMS-FAB^+^: *m*/*z* [*M*+H]^+^ calcd for C_20_H_27_O_3_: 315.1960, found: 315.1975; Anal. calcd for C_20_H_26_O_3_: C 76.39, H 8.34, found: C 76.1, H 8.41. The mother liquor of the crystals obtained above was fractionated by flash chromatography (CHCl_3_/EtOAc, 8:1 → 2:1 gradient) to yield another 150 mg of **10 b** as white residue.

**6-Oxo-6,7,8,9,10,11,12,13,14,15,16,17-dodecahydrocyclotrideca[*c*]chromen-3-yl sulfamate (10).** Compound **10 b** (370 mg, 1.18 mmol) was sulfamoylated in a similar manner to **6 b** and the crude light-yellow residue obtained (407 mg) on dissolving in a minimal volume of acetone was fractionated by flash chromatography (CHCl_3_/acetone, 12:1 → 2:1 gradient). The second fraction that was isolated gave a white residue (140 mg, 20 %) which was recrystallised from THF/hexane to give **10** as white crystals (74 mg): *R*_f_=0.34 (CHCl_3_/acetone, 8:1); mp: 170–174 °C; ^1^H NMR (400 MHz, [D_6_]DMSO): *δ*=1.2–1.7 (18 H), 2.47 (m, 2 H, C17-CH_2_), 7.26 (dd, *J*=2.3 and 8.7 Hz, 1 H, C2-H), 7.30 (d, *J*=2.3 Hz, 1 H, C4-H), 7.92 (d, *J*=8.7 Hz, 1 H, C1-H) and 8.20 ppm (s, 2 H, ex. with D_2_O, NH_2_); MS (FAB^+^): *m*/*z* (%): 787.1 (6) [2 *M*+H]^+^, 394.0 (100) [*M*+H]^+^; MS (FAB^−^): *m*/*z* (%): 785.2 (12) [2 *M*−H]^−^, 392.1 (100) [*M*−H]^−^, 313.2 (50) [*M*−H_2_NSO_2_]^−^; HRMS-FAB^+^: *m*/*z* [*M*+H]^+^ calcd for C_22_H_28_NO_5_S: 394.1668, found, 394.1712; Anal. calcd for C_20_H_27_NO_5_S: C 61.04, H 6.92, N 3.56, found C 61.4, H 7.22, N 3.27.

**Ethyl 2-oxocyclopentadecanecarboxylate (11 a).** Prepared in a similar manner to **6 a** using NaH (891 mg, 22.3 mmol), diethyl carbonate (70 mL) and cyclopentadecanone (2.5 g, 11.2 mmol). The crude yellow syrup obtained was purified by flash chromatography (CH_2_Cl_2_) to give **11 a** as a pale-yellow oil (1.62 g, 49 %): *R*_f_=0.70 (CH_2_Cl_2_); ^1^H NMR (400 MHz, CDCl_3_): *δ*=1.64 (t, *J*=6.7 Hz, 3 H, CH_2_C*H*_3_), 1.15–1.62 (m, 25 H), 2.55 (t, *J*=7.0 Hz, 2 H, ring C2-CH_2_) and 4.16 ppm (q, *J*=7.3 Hz, 2 H, C*H*_2_CH_3_); MS (FAB^+^): *m*/*z* (%): 297.2 (100) [*M*+H]^+^; MS (FAB^−^): *m*/*z* (%): 295.2 (100) [*M*−H]^−^; HRMS-FAB^+^: *m*/*z* [*M*+H]^+^ calcd for C_18_H_33_O_3_: 297.2429, found: 297.2430.

**3-Hydroxy-8,9,10,11,12,13,14,15,16,17,18,19-dodecahydrocyclopentadeca[*c*]chromen-6(7*H*)-one (11 b).** Prepared in a similar manner to **6 b** using resorcinol (558 mg, 5.06 mmol), **11 a** (1.5 g, 5.1 mmol) and a mixture of CF_3_COOH (1.0 mL, 10 mmol) and concd H_2_SO_4_ (1.0 mL, 10 mmol). The crude brown solid obtained was purified by flash chromatography (CHCl_3_/acetone, 8:1 → 4:1 gradient) and the yellow solid that was isolated was recrystallised from THF/hexane to give **11 b** as pale-yellow crystals (432 mg, 25 %): *R*_f_=0.69 (CHCl_3_/acetone, 3:1); mp: 209–211 °C; ^1^H NMR (400 MHz, CDCl_3_): *δ*=1.25–1.62 (m, 22 H, 11×CH_2_), 2.57 (t, *J*=7.8 Hz, 2 H, C7-CH_2_), 2.74 (t, *J*=7.0 Hz, 2 H, C19-CH_2_), 6.04 (s, 1 H, OH), 6.81 (dd, *J*=2.7 and 8.9 Hz, 1 H, C2-H), 6.92 (d, *J*=2.7 Hz, 1 H, C4-H) and 7.45 ppm (d, *J*=8.9 Hz, 1 H, C1-H); MS (FAB^+^): *m*/*z* (%): 343.1 (100) [*M*+H]^+^; MS (FAB^−^): *m*/*z* (%): 341.2 (100) [*M*−H]^−^; HRMS-FAB^+^: *m*/*z* [*M*+H]^+^ calcd for C_22_H_31_O_3_: 343.2273, found: 343.2269; Anal. calcd for C_22_H_30_O_3_: C 77.16, H, 8.83, found C 77.12, H 8.89; HPLC: Waters Radialpak column, MeOH/H_2_O (90:10), flow rate=2 mL min^−1^, *λ*_max_=324.4 nm, *t*_R_=8.5 min, purity >98 %.

**6-Oxo-6,7,8,9,10,11,12,13,14,15,16,17,18,19-tetradecahydrocyclopentadeca[*c*]chromen-3-yl sulfamate (11).** Compound **11 b** (350 mg, 1.02 mmol) was sulfamoylated in a similar manner to **6 b** and the crude white solid obtained was purified by flash chromatography (CHCl_3_/EtOAc, 8:1 → 2:1 gradient) to give a thick waxy solid that was difficult to recrystallise. Further purification by preparative TLC (CHCl_3_/EtOAc, 4:1) gave a white solid (201 mg), that was recrystallised from THF/hexane to give **11** as fine white flakes (185 mg, 43 %): *R*_f_=0.50 (CHCl_3_/EtOAc, 4:1); mp: 163–166 °C; ^1^H NMR (400 MHz, [D_6_]DMSO): *δ*=1.32–1.59 (m, 22 H, 11×CH_2_), 2.51–2.81 (m, 4 H, C7-CH_2_ and C19-CH_2_), 7.26–7.28 (m, 2 H, C2-H and C4-H), 7.89 (d, *J*=7.8 Hz, 1 H, C1-H) and 8.19 ppm (s, 2 H, NH_2_); MS (FAB^+^): *m*/*z* (%): 842.3 (70) [2 *M*+H]^+^, 422.1 (100) [*M*+H]^+^; MS (FAB^−^): *m*/*z* (%): 841.4 (80) [2 *M*−H]^−^, 420.2 (100) [*M*−H]^−^, 341.2 (60) [*M*−H_2_NSO_2_]^−^; HRMS-FAB^+^: *m*/*z* [*M*+H]^+^ calcd for C_22_H_32_NO_5_S: 422.1999, found: 422.1994; Anal. calcd for C_22_H_31_NO_5_S: C 62.68, H 7.41, N 3.32, found: C 62.80, H 7.56, N 3.00; HPLC: Waters Radialpak column, MeOH/H_2_O (90:10), flow rate=2 mL min^−1^, *λ*_max_=285.2 and 313.7 nm, *t*_R_=4.2 min, purity >98 %.

**6-Oxo-6,7,8,9,10,11-hexahydrocyclohepta[*c*]chromen-3-yl dimethylsulfamate (12).** *N*,*N*-Dimethylsulfonyl chloride (1.90 mL, 17.55 mmol) was added dropwise to a mixture of 3-hydroxy-8,9,10,11-tetrahydrocyclohepta[*c*]chromen-6(7*H*)-one (2.0 g, 8.69 mmol)[17] in *N*,*N*-dimethylcyclohexylamine (10 mL). The resulting mixture was heated at 90 °C for 1 h. The brown slurry obtained was cooled to room temperature and diluted with EtOAc (150 mL). The organic fraction was then washed sequentially with NaOH (1 m, 2×100 mL), HCl (2 m, 2×100 mL) and brine (3×50 mL); it was dried (MgSO_4_) and evaporated to give a light-yellow residue (2.94 g). Recrystallisation from hot EtOAc/hexane (2.5:1) gave **12** as a light-yellow crystalline solid (2.0 g, 5.93 mmol, 68 %): mp: 159–160.5 °C; ^1^H NMR (400 MHz, [D_6_]DMSO): *δ*=1.52 (m, 2 H), 1.61 (m, 2 H), 1.86 (m, 2 H), 2.81 (m, 2 H), 2.94 (s, 6 H, 2×N-CH_3_), 3.00 (m, 2 H, C11-H_2_), 7.32 (dd, *J*=2 and 8.9 Hz, 1 H, C2-H), 7.39 (d, *J*=2.73 Hz, 1 H, C4-H) and 8.01 ppm (d, *J*=8.9 Hz, 1 H, C1-H); MS (AP^+^): *m*/*z* (%): 338.2 (100) [*M*+H]^+^; Anal. calcd for C_16_H_19_NO_5_S: C 56.96, H 5.68, N, 4.15, found: C 57.0, H 5.71, N 4.32.

**4-Methoxybenzene-1,3-diol (13 a).** Starting material **13 a** was prepared according to the method of Godfrey et al.[20]

**3-Hydroxy-2-methoxy-8,9,10,11-tetrahydrocyclohepta[*c*]chromen-6(7*H*)-one (13 b).** A mixture of **13 a** (1.05 g, 7.49 mmol) and methyl 2-oxo-1-cycloheptane carboxylate (1.35 g, 7.87 mmol) at 0 °C was treated dropwise with a mixture of CF_3_COOH (1.2 mL, 15 mmol) and concd H_2_SO_4_ (1.5 mL, 15 mmol) while keeping the reaction temperature <10 °C. After stirring for 3 h at room temperature, the dark-brown mixture was cautiously quenched with ice-water followed by the addition of EtOAc (200 mL). The organic layer that separated was washed with H_2_O (4×100 mL) and dried by azeotropic evaporation with *i*PrOH. The dark-purple residue obtained (2.0 g) was recrystallised from hot EtOAc and hexane to give **13 b** as pink crystals (1.23 g, 25 %): mp: 158–159 °C. Upon fractionation of the residue retrieved from the mother liquor by flash chromatography (EtOAc/hexane, 1:4 → 4:1 gradient), the second fraction that was isolated gave a yellow residue (321 mg) that was recrystallised from hot EtOAc and hexane to give a second crop of **13 b** (184 mg, total 72 %) as creamy crystals: *R*_f_=0.46 (EtOAc/hexane, 2:1); mp: 158–159 °C; ^1^H NMR (400 MHz, [D_6_]DMSO): *δ*=1.49 (m, 2 H), 1.60 (m, 2 H), 1.83 (m, 2 H), 2.76 (m, 2 H, C7-H_2_), 2.96 (m, 2 H, C11-CH_2_), 3.86 (s, 3 H, OCH_3_), 6.76 (s, 1 H, C4-H), 7.25 (s, 1 H, C1-H) and 10.15 ppm (br s, 1 H, ex. with D_2_O, OH); MS (FAB^+^): *m*/*z* (%) 259.1 (100) [*M*+H]^+^; MS (FAB^−^): *m*/*z* (%): 257.1 (100) [*M*−H]^−^; HRMS-FAB^+^: *m*/*z* [*M*+H]^+^ calcd for C_16_H_19_O_3_: 259.1334, found: 259.1323; Anal. calcd for C_15_H_16_O_4_: C 69.20, H 6.20, found: C 69.1, H 6.16.

**2-Methoxy-6-oxo-6,7,8,9,10,11-hexahydrocyclohepta[*c*]chromen-3-yl sulfamate (13).** Compound **13 b** (500 mg, 1.92 mmol) in anhydrous DMF (10 mL) was sulfamoylated in a similar manner to **6 b**. The crude pale-yellow residue obtained was purified by flash chromatography (CHCl_3_/THF, 16:1 → 2:1 gradient). The second fraction isolated gave a white solid that was recrystallised from THF/hexane to give **13** as a white powder (204 mg, 31 %): mp: 193–195 °C; ^1^H NMR (400 MHz, [D_6_]DMSO): *δ*=1.52 (m, 2 H), 1.62 (m, 2 H), 1.86 (m, 2 H), 2.83 (m, 2 H, C7-H_2_), 3.04 (m, 2 H, C11-CH_2_), 3.91 (s, 3 H, OCH_3_), 7.37 (s, 1 H, C4-H), 7.48 (s, 1 H, C1-H) and 8.17 ppm (s, 2 H, ex. with D_2_O, OSO_2_NH_2_); MS (ES^+^) *m*/*z* (%): 340.0 (100) [*M*+H]^+^; Anal. calcd for C_15_H_17_NO_6_S: C 53.09, H 5.05, N 4.13, found C 53.1, H 5.06, N 4.01; HPLC: Sunfire C_18_ reversed-phase column, 4.6×75 mm, 3.5 μm pore size, MeOH/H_2_O (80:20), flow rate=0.8 mL min^−1^, *t*_R_=2.12 min, purity >98 %.

**2-hydroxy-8,9,10,11-tetrahydrocyclohepta[*c*]chromen-6(7*H*)-one (14 a).** Hydroquinone (3.56 g, 32.34 mmol) was dissolved in hot methyl 2-oxo-1-cycloheptanecarboxylate (5.0 g, 4.59 mL, 29.4 mmol). To this stirred brown suspension at ice-water temperature was added dropwise a mixture of CF_3_COOH (5.0 mL, 64.68 mmol) and concd H_2_SO_4_ (6.47 mL, 64.68 mmol) at such a rate that the reaction temperature was kept <10 °C (∼30 min). The reaction mixture was then allowed to warm to room temperature and thereupon stirred for an additional 60 h before being quenched cautiously with ice-water. After stirring the suspension that formed for 1 h, the pale-cream precipitate was collected by suction filtration, washed exhaustively with H_2_O and air dried. The crude product was purified by recrystallisation from acetone to give **14 a** as colourless needles (0.19 g, 3 %): mp: 204–206 °C; ^1^H NMR (270 MHz, [D_6_]DMSO): *δ*=1.49 (m, 2 H), 1.69 (m, 2 H), 1.79 (m, 2 H), 2.68 (m, 2 H), 2.83 (m, 2 H), 7.16 (dd, *J*=2.95 and 8.9 Hz, 1 H), 7.30 (d, *J*=2.9 Hz, 1 H), 7.43 (d, *J*=8.9 Hz, 1 H), 9.88 ppm (s, 1 H, ex. with D_2_O); ^13^C NMR (100 MHz, [D_6_]DMSO): *δ*=21.65 (CH_2_), 24.48 (CH_2_), 26.11 (CH_2_), 31.29 (CH_2_), 34.01 (CH_2_), 107.80 (CH), 119.32 (CH), 120.82, 122.41 (CH), 123.08, 148.92, 154.30, 168.69, 175.45 ppm; MS (FAB^+^): *m*/*z* (%): 231.1 (100) [*M*+H]^+^; HRMS-FAB^+^: *m*/*z* [*M*+H]^+^ calcd for C_14_H_15_O_3_: 231.10212, found 231.10255; Anal. calcd for C_14_H_15_O_3_: C 73.0, H 6.13, found: C 73.0, H 6.15.

**6-oxo-6,7,8,9,10,11-hexahydrocyclohepta[*c*]chromen-2-yl sulfamate (14).** To an ice-cooled solution of **14 a** (100 mg, 0.43 mmol) in anhydrous DMA (5 mL) was added sulfamoyl chloride (0.7 m solution in toluene, 3.04 mL; the toluene was removed in vacuo [not allowing the temperature of the water bath to exceed 30 °C] prior to addition, 4.34 mmol) and the mixture stirred (under a positive flow of dry N_2_) overnight. The mixture was diluted with EtOAc (25 mL), washed with H_2_O (3×50 mL) and brine (50 mL) and concentrated in vacuo (not allowing the temperature of the water bath to exceed 30 °C). The product was precipitated with Et_2_O/ *n*-hexane, washed with *n*-hexane, and vacuum dried to give **14** as an off-white amorphous powder (70 mg, 52 %): mp: 185–187 °C (dec); ^1^H NMR (400 MHz, [D_6_]DMSO): *δ*=1.50 (m, 2 H), 1.69 (m, 2 H), 1.81 (m, 2 H), 2.70 (m, 2 H), 2.88 (m, 2 H), 7.59 (dd, *J*=3.2 and 8.97 Hz, 1 H), 7.68 (d, *J*=8.97 Hz, 1 H), 7.86 (d, *J*=3.2 Hz, 1 H), 8.08 ppm (s, 2 H, ex. with D_2_O); ^13^C NMR (100 MHz, [D_6_]DMSO): *δ*=22.49 (CH_2_), 25.29 (CH_2_), 26.79 (CH_2_), 32.05 (CH_2_), 34.81 (CH_2_), 118.54, 120.79, 122.58 (CH), 123.39 (CH), 128.90 (CH), 147.27, 153.73, 170.07, 175.55 ppm; MS (FAB^+^) *m*/*z* (%): 310.1 (100) [*M*+H]^+^; HRMS-FAB^+^: *m*/*z* [*M*+H]^+^ calcd for C_14_H_16_NSO_5_: 310.0749, found: 310.0753; Anal. calcd for C_14_H_16_NSO_5_: C 54.4, H 4.89, N 4.53, found: C 54.0, H 5.01, N 4.31; purity of sample (as calculated by ^1^H NMR): 97.4 %

**3-Hydroxy-8,9,10,11-tetrahydro-5*H*-cyclohepta[*c*]quinolin-6(7*H*)-one (16 a).** A slurry of 3-aminophenol (2.0 g, 18.33 mmol) in methyl 2-oxo-1-cycloheptane carboxylate (3.12 g, 18.33 mmol) was heated at 150 °C for 8 h. After cooling, EtOAc (50 mL) was added to the crude dark-brown residue and the resulting suspension was triturated in an ultrasonic bath for 30 min followed by filtration. The precipitate that collected was washed with more EtOAc and air dried to give **16 a** as pink/light-brown residue (3.05 g, 13.30 mmol, 73 %): mp: 290–300 °C; ^1^H NMR (400 MHz, [D_6_]DMSO): *δ*=1.44 (m, 2 H), 1.55 (m, 2 H), 1.81 (m, 2 H), 2.81 (m, 2 H, C7-H_2_), 2.93 (m, 2 H, C11-H_2_), 6.62 (dd, *J*=2.1 and 8.7 Hz, 1 H, C2-H), 6.69 (d, *J*=2.1 Hz, 1 H, C4-H), 7.64 (d, *J*=8.7 Hz, 1 H, C1-H), 10.1 (s, 1 H, ex. with D_2_O, OH) and 11.4 ppm (1 H, s, ex. with D_2_O, NH); MS (FAB^+^) *m*/*z* (%) 230.3 (100) [*M*+H]^+^; MS (FAB^−^) *m*/*z* (%) 382.3 (45) [*M*+NBA]^−^, 228.3 (100) [*M*−H]^−^; HRMS-FAB^+^: *m*/*z* [*M*+H]^+^ calcd for C_14_H_16_NO_2_: 230.1181, found: 230.1184. This crude product was used for the next reaction without further purification.

**6-Oxo-6,7,8,9,10,11-hexahydro-5*H*-cyclohepta[*c*]quinolin-3-yl sulfamate (16).** NaH (60 % in mineral oil, 53 mg, 1.31 mmol) was added to a solution of **16 a** (300 mg, 1.30 mmol) in anhydrous DMF (5 mL) at 0 °C, followed by a concentrated solution of sulfamoyl chloride (∼0.69 m in toluene, ∼5 equiv) in one portion 15 min later after the evolution of H_2_ had ceased. The reaction mixture was stirred at room temperature under an atmosphere of N_2_ overnight before diluting with EtOAc (100 mL). The resulting mixture was washed with brine (4×50 mL), dried (MgSO_4_) and concentrated in vacuo to give an off-white residue that was fractionated on silica with EtOAc. The first fraction that was collected gave an off-white syrup (284 mg), which upon crystallisation from EtOAc/hexane (5:1) gave **16** as white crystals (174 mg, 564 μmol, 43 %): mp: 180–185 °C; IR (KBr) 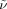
=3420, 3300, 3200–3000, 2920, 2860, 1630, 1550, 1380, 1180 cm^−1^; ^1^H NMR (270 MHz, [D_6_]DMSO): *δ*=1.46 (m, 2 H), 1.57 (m, 2 H), 1.85 (m, 2 H), 2.87 (m, 2 H, C7-H_2_), 3.02 (m, 2 H, C11-H_2_), 7.08 (dd, *J*=2.4 and 9 Hz, 1 H, C2-H), 7.23 (d, *J*=2.2 Hz, 1 H, C4-H), 7.94 (d, *J*=8.8 Hz, 1 H, C1-H), 8.10 (s, 2 H, ex. with D_2_O, OSO_2_NH_2_) and 11.8 ppm (s, 1 H, ex. with D_2_O, NH); MS (FAB^+^): *m*/*z* (%): 309.2 (100) [*M*+H]^+^, 230.2 (12) [*M*−H_2_NSO_2_]^+^; HRMS-FAB^+^: *m*/*z* [*M*+H]^+^ calcd for C_14_H_17_N_2_O_4_S: 309.0909, found: 309.0916; Anal. calcd for C_14_H_16_N_2_O_4_S: C 54.53, H 5.23, N 9.08, found: C 54.7, H 5.27, N 8.96.

**3-(Benzyloxy)-8,9,10,11-tetrahydro-5*H*-cyclohepta[*c*]quinolin-6(7*H*)-one (17).** NaH (60 % in mineral oil, 350 mg, 8.75 mmol) was added to a solution of **16 a** (2.0 g, 8.72 mmol) in DMF (100 mL) at 0 °C, followed by benzyl bromide (1.1 mL, 9.33 mmol) 15 min later after the evolution of H_2_ had ceased. The reaction mixture was heated at 90 °C for 30 min and then concentrated in vacuo after cooling to room temperature. The light-beige sludge that was obtained was diluted with EtOAc (200 mL) and filtered. The precipitate that collected was washed with more EtOAc and H_2_O (4×50 mL) and air dried overnight to give **17** as a white powder (2.2 g, 6.89 mmol, 79 %): *R*_f_=0.69 (CHCl_3_/acetone, 1:2), c.f. *R*_f_=0.58 (**16 a**); IR (KBr) 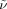
=3000–2800, 1650 cm^−1^; ^1^H NMR (400 MHz, [D_6_]DMSO): *δ*=1.42 (m, 2 H), 1.53 (m, 2 H), 1.81 (m, 2 H), 2.80 (m, 2 H), 2.94 (m, 2 H), 5.11 (s, 2 H, OCH_2_), 6.83 (dd, *J*=2.7 and 9 Hz, 1 H, C2-H), 6.87 (d, *J*=2.7 Hz, 1 H, C4-H), 7.38 (m, 5 H, Ph), 7.73 (d, *J*=9 Hz, 2 H, C1-H) and 11.5 ppm (s, 1 H, ex. with D_2_O, NH); MS (FAB^+^): *m*/*z* (%): 320.0 (100) [*M*+H]^+^, 229.0 (5) [*M*+H−Bn]^+^, 91.0 (42) [Bn]^+^; HRMS-FAB^+^: *m*/*z* [*M*+H]^+^ calcd for C_21_H_22_NO_2_: 320.1651, found: 320.1661. This crude product was used for the next reaction without further purification.

**3-(Benzyloxy)-5-methyl-8,9,10,11-tetrahydro-5*H*-cyclohepta[*c*]quinolin-6(7*H*)-one (18 a).** NaH (60 % in mineral oil, 65 mg, 1.63 mmol) was added to a solution of **17** (500 mg, 1.57 mmol) in DMF (130 mL) at 0 °C, followed by CH_3_I (0.2 mL, 3.15 mmol) 15 min later after the evolution of H_2_ had ceased. The reaction mixture was heated at 80 °C for 50 min and then concentrated in vacuo after cooling to room temperature. The beige residue obtained was dissolved in EtOAc (150 mL), and the resulting mixture washed with brine (3×50 mL), dried (MgSO_4_), filtered and evaporated to give a yellow residue. This crude product was fractionated on silica with CHCl_3_/EtOAc (8:1 → 2:1 gradient). The second fraction that was collected upon evaporation gave **18 a** as a white residue (480 mg, 1.44 mmol, 92 %). An analytical sample of this residue was recrystallised from EtOAc/hexane (1:2) to give **18 a** as soft fine needle-shaped crystals: mp: 119–121 °C; *R*_f_=0.78 (CHCl_3_/EtOAc, 1:2), c.f. *R*_f_=0.58 (**17**); IR (KBr) 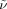
=2920, 2840, 1630, 1610, 1590, 1240 cm^−1^; ^1^H NMR (400 MHz, [D_6_]DMSO): *δ*=1.45 (m, 2 H), 1.56 (m, 2 H), 1.83 (m, 2 H), 2.90 (m, 2 H), 2.98 (m, 2 H), 3.61 (s, 3 H, NCH_3_), 5.26 (s, 2 H, OCH_2_), 6.96 (dd, *J*=2.3 and 8.9 Hz, 1 H, C2-H), 7.04 (d, *J*=2.7 Hz, 1 H, C4-H), 7.43 (m, 5 H, Ph) and 7.86 ppm (d, *J*=8.9 Hz, 1 H, C1-H); ^13^C NMR (100.4 MHz, CDCl_3_) *δ*=26.01 (t), 26.54 (t), 26.91 (t), 28.64 (t), 30.68 (q, NCH_3_), 32.59 (t), 70.62 (t, OCH_2_), 100.36 (d), 109.93 (d), 115.12 (s), 126.02 (d), 127.65 (d), 128.38 (d), 128.87 (d), 130.77 (s), 136.55 (s), 140.45 (s), 148.84 (s), 159.86 (s) and 162.57 ppm (s); MS (FAB^+^): *m*/*z* (%): 334.3 (100) [*M*+H]^+^, 243.2 (5) [*M*+H−Bn]^+^, 91.1(35) [Bn]^+^; HRMS-FAB^+^: *m*/*z* [*M*+H]^+^ calcd for C_22_H_24_NO_2_: 334.1807, found: 334.1798; Anal. calcd for C_22_H_23_NO_2_: C 79.25, H 6.95, N 4.20, found: C 79.5, H 7.00, N 4.27.

**3-Hydroxy-5-methyl-8,9,10,11-tetrahydro-5*H*-cyclohepta[*c*]quinolin-6(7*H*)-one (18 b).** Compound **18 a** (460 mg, 1.38 mmol) in THF (15 mL) was added to a suspension of Pd/C (10 %, 100 mg) in THF (15 mL). The reaction mixture was stirred under an atmosphere of H_2_ (balloon) at room temperature, and the progress of the reaction was monitored by TLC. After the disappearance of the starting material had completed, the suspension was filtered and the charcoal retained washed with more THF. The combined filtrates were concentrated in vacuo, and the light-yellow residue obtained was recrystallised from hot THF/hexane (1:1) to give **18 b** as fine pale-yellow crystals (148 mg, 608 μmol, 44 %): *R*_f_=0.37 (CHCl_3_/EtOAc, 1:2), c.f. *R*_f_=0.63 (**18 a**); mp: 255–261 °C; ^1^H NMR (400 MHz, [D_6_]DMSO): *δ*=1.45 (m, 2 H), 1.56 (m, 2 H), 1.82 (m, 2 H), 2.88 (m, 2 H), 2.97 (m, 2 H), 3.56 (s, 3 H, NCH_3_), 6.73 (dd, *J*=2.3 and 9 Hz, 1 H, C2-H), 6.78 (d, *J*=2 Hz, 1 H, C4-H), 7.76 (d, *J*=9 Hz, 1 H, C1-H) and 10.11 ppm (br s, 1 H, ex. with D_2_O, OH); MS (FAB^+^): *m*/*z* (%): 244.2 (100) [*M*+H]^+^; MS (FAB^−^): *m*/*z* (%): 396.3 (43) [*M*+NBA]^−^, 242.2 (100) [*M*−H]^−^; HRMS-FAB^+^: *m*/*z* [*M*+H]^+^ calcd for C_15_H_18_NO_2_: 244.1338, found: 244.1333.

**5-Methyl-6-oxo-6,7,8,9,10,11-hexahydro-5*H*-cyclohepta[*c*]quinolin-3-yl sulfamate (18).** Compound **18 b** (100 mg, 411 μmol) in anhydrous DMF (5 mL) was sulfamoylated in a similar manner to **16 a**. The crude pale-yellow syrup (130 mg) obtained was fractionated on silica with EtOAc, and the first fraction that was collected gave a pale-yellow syrup which upon crystallisation from hot EtOAc/hexane (1:2) gave **18** as white crystals (45 mg, 140 μmol, 34 %): *R*_f_=0.78 (EtOAc), c.f. *R*_f_=0.68 (**18 b**); mp: 185–187 °C; ^1^H NMR (400 MHz, [D_6_]DMSO): *δ*=1.47 (m, 2 H), 1.58 (m, 2 H), 1.85 (m, 2 H), 2.95 (m, 2 H), 3.06 (m, 2 H), 3.63 (s, 3 H, NCH_3_), 7.19 (dd, *J*=2.2 and 9 Hz, 1 H, C2-H), 7.37 (d, *J*=2.2 Hz, 1 H, C4-H), 7.76 (d, *J*=9.2 Hz, 1 H, C1-H) and 8.11 ppm (s, 2 H, ex. with D_2_O, OSO_2_NH_2_); MS (FAB^+^): *m*/*z* (%): 323.1 (100) [*M*+H]^+^, 243.1 (10) [*M*−HNSO_2_]^+^; MS (FAB^−^): *m*/*z* (%): 321.1(100) [*M*−H)^−^, 242.1 (12) [*M*−H_2_NSO_2_]^−^; HRMS-FAB^+^: *m*/*z* [*M*+H]^+^ calcd for C_15_H_19_N_2_O_4_S: 323.1066, found: 323.1054; Anal. calcd for C_15_H_18_N_2_O_4_S: C 55.89, H 5.63, N 8.69, found: C 55.8, H 5.63, N, 8.63.

**3-(Benzyloxy)-6-chloro-8,9,10,11-tetrahydro-7*H*-cyclohepta[*c*]quinoline (19 a).** A suspension of **17** (1.0 g, 3.13 mmol) in POCl_3_ (20 mL) was held at reflux for 2 h. After cooling to room temperature, ice-water and EtOAc (100 mL) were added to the dark-red/brown reaction mixture. The organic layer that separated was washed with H_2_O (4×50 mL), dried (MgSO_4_), filtered and concentrated in vacuo to give a light-yellow residue. This crude product was recrystallised from hot *i*PrOH to give **19 a** as light-yellow crystals (840 mg, 2.49 mmol, 79 %): *R*_f_=0.60 (EtOAc/hexane, 1:2), c.f. *R*_f_<0.05 (**17**); mp: 128.5–130.5 °C; ^1^H NMR (400 MHz, [D_6_]DMSO) *δ*=1.62 (m, 4 H), 1.87 (m, 2 H), 3.16 (m, 2 H), 3.29 (m, 2 H), 5.28 (s, 2 H, OCH_2_), 7.41 (m, 7 H) and 8.16 ppm (d, *J*=7.4 Hz, 1 H); MS (FAB^+^): *m*/*z* (%) 338.3 (100) [*M*+H]^+^, 91.1 (55); HRMS-FAB^+^: *m*/*z* [*M*+H]^+^ calcd for C_21_H_21_NO^35^Cl: 338.1312, found: 338.1308; Anal. calcd for C_21_H_20_NOCl: C 74.66, H 5.97, N 4.15, found: C 74.5, H 5.94, N 4.22.

**3-(Benzyloxy)-6-methoxy-8,9,10,11-tetrahydro-7*H*-cyclohepta[*c*]quinoline (19 b).** NaH (60 % in mineral oil, 296 mg, 7.40 mmol) was added to a mixture of anhydrous MeOH (240 mg, 7.49 mmol) and anhydrous DMF (15 mL) at ice-water temperature. After stirring for 15 min, the resulting purple–grey mixture was then transferred dropwise through a cannula to a solution of **19 a** (500 mg, 1.48 mmol) in anhydrous DMF (10 mL). The brown mixture/suspension that resulted was heated at 70 °C for 2 h, cooled, and diluted with EtOAc (150 mL). The organic fraction was washed with brine (5×100 mL), dried (MgSO_4_), filtered and concentrated in vacuo to give a yellow–brown residue that was fractionated on silica with EtOAc/hexane (1:8 → 1:4 gradient). The first fraction that collected upon evaporation gave **19 b** as a white residue (387 mg, 1.16 mmol, 78 %): *R*_f_=0.60 (EtOAc/hexane, 1:4), c.f. *R*_f_=0.51 (**19 a**); ^1^H NMR (400 MHz, [D_6_]DMSO) *δ*=1.52 (m, 4 H), 1.60 (m, 2 H), 1.86 (m, 2 H), 2.95 (m, 2 H), 3.16 (m, 2 H), 3.95 (s, 3 H, OCH_3_), 5.25 (s, 2 H, OCH_2_), 7.11 (dd, *J*=2.6 and 9.2 Hz, 1 H, C2-H), 7.24 (d, *J*=2.6 Hz, 1 H, C4-H), 7.41 (m, 5 H, Ph) and 7.99 ppm (d, *J*=9.2 Hz, 1 H, C1-H); MS (FAB^+^): *m*/*z* (%): 334.3 (100) [*M*+H]^+^, 91.1 (64); HRMS-FAB^+^: *m*/*z* [*M*+H]^+^ calcd for C_22_H_24_NO_2_: 334.1807, found: 334.1801.

**6-Methoxy-8,9,10,11-tetrahydro-7*H*-cyclohepta[*c*]quinolin-3-ol (19 c).** A solution of **19 b** (689 mg, 2.07 mmol) in absolute EtOH (70 mL) was debenzylated by hydrogenation in a manner similar to **18 a** in the presence of Pd/C (10 %, 70 mg). The crude light-yellow residue that resulted (422 mg) was recrystallised from CHCl_3_/hexane (5:6) to give **19 c** as white crystals (253 mg, 1.04 mmol, 50 %): *R*_f_=0.38 (CHCl_3_/EtOAc, 4:1), c.f. *R*_f_=0.79 (**19 b**); mp: undefined but all melted by 177 °C; IR (KBr) 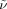
=3600–2500, 2910, 2840, 1620, 1240 cm^−1^; ^1^H NMR (400 MHz, [D_6_]DMSO) *δ*=1.51 (m, 2 H), 1.58 (m, 2 H), 1.85 (m, 2 H), 2.92 (m, 2 H), 3.12 (m, 2 H), 3.93 (s, 3 H, OCH_3_), 6.92 (dd, *J*=2.5 and 9.2 Hz, 1 H, C2-H), 7.00 (d, *J*=2.3 Hz, 1 H, C4-H), 7.89 (d, *J*=9.3 Hz, 1 H, C1-H) and 9.84 ppm (br s, 1 H, ex. with D_2_O, OH); MS (FAB^+^) *m*/*z* (%) 244.3 (100) [*M*+H]^+^; MS (FAB^−^) *m*/*z* (%) 395.4 (23) [*M*−H+NBA]^−^, 242.3 (100) [*M*−H]^−^; HRMS- FAB^+^: *m*/*z* [*M*+H]^+^ calcd for C_15_H_18_NO_2_: 244.1338, found: 244.1333; Anal. calcd for C_15_H_17_NO_2_: C 74.05, H 7.04, N 5.76, found: C 73.8, H 7.01, N 5.82.

**6-Methoxy-8,9,10,11-tetrahydro-7*H*-cyclohepta[*c*]quinolin-3-yl sulfamate (19).** Sulfamoyl chloride (∼5 equiv) was added to a solution of **19 c** (70 mg, 288 μmol) and 2,6-di-*tert*-butyl-4-methylpyridine (60 mg, 292 μmol) in anhydrous CH_2_Cl_2_ (10 mL) at room temperature. After stirring for 5 h under an atmosphere of N_2_, the reaction mixture was concentrated in vacuo and the resulting yellow syrup was dissolved in EtOAc (50 mL). The organic fraction was washed with HCl (0.5 m, 4×25 mL), H_2_O (2×50 mL), dried (MgSO_4_) and concentrated in vacuo to give a light-brown residue (114 mg) that upon recrystallisation from CHCl_3_/hexane (2:5) gave **19** as white crystals (32 mg, 99.3 μmol, 34 %): *R*_f_=0.30 (EtOAc/hexane), c.f. *R*_f_=0.36 (**19 c**); mp=94–97 °C; IR (KBr) 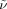
=3540, 3350, 3240, 2910, 2840, 1360, 1180, 1170 cm^−1^; ^1^H NMR (400 MHz, [D_6_]DMSO): *δ*=1.54 (m, 2 H), 1.61 (m, 2 H), 1.89 (m, 2 H), 3.00 (m, 2 H), 3.23 (m, 2 H), 3.99 (s, 3 H, OCH_3_), 7.31 (dd, *J*=2.3 and 9 Hz, 1 H, C2-H), 7.63 (d, *J*=2.3 Hz, 1 H, C4-H), 8.07 (br s, 2 H, ex. with D_2_O, OSO_2_NH_2_) and 8.18 ppm (d, *J*=9 Hz, 1 H, C1-H); MS (FAB^+^): *m*/*z* (%): 323.3 (8) [*M*+H]^+^, 309.2 (100) [*M*+H−CH_2_]^+^, 230.2 (13) [*M*+H−CH_2_−HNSO_2_]^+^; HRMS-FAB^+^: *m*/*z* [*M*+H−CH_2_]^+^ calcd for C_14_H_17_N_2_O_4_S: 309.0909, found: 309.0914; Anal. calcd for C_15_H_18_N_2_O_4_S: C 55.89, H 5.63, N 8.69, found: C 54.9, H 5.71, N 8.63.

**3-(Benzyloxy)-5-pentyl-8,9,10,11-tetrahydro-5*H*-cyclohepta[*c*]quinolin-6(7*H*)-one (20 a) and 3-(benzyloxy)-6-(pentyloxy)-8,9,10,11-tetrahydro-7*H*-cyclohepta[*c*]quinoline (21 a).** NaH (60 % in mineral oil, 65 mg, 1.62 mmol) was added to a solution of **17** (500 mg, 1.57 mmol) in DMF (40 mL) at room temperature cautiously followed by 1-bromopentane (0.4 mL, 3.23 mmol) 15 min later after the evolution of H_2_ had ceased. The reaction mixture was heated at 100 °C for 1 h and then concentrated in vacuo after cooling to room temperature. The crude material that obtained was dissolved in EtOAc (100 mL) and the resulting mixture was washed with brine (4×50 mL), dried (MgSO_4_), filtered and evaporated to give a yellow syrup which was fractionated on silica eluting first with CHCl_3_/hexane (4:1), then CHCl_3_ followed by CHCl_3_/EtOAc (4:1 → 1:1 gradient). The first fraction that collected upon evaporation gave **21 a** as a white residue (250 mg, 642 μmol, 41 %): *R*_f_=0.73 (CHCl_3_/hexane, 2:1), c.f. *R*_f_<0.05 (**17**); IR (KBr) 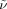
=3000–2840, 1615, 1590, 1330 cm^−1^; ^1^H NMR (400 MHz, [D_6_]DMSO): *δ*=0.90 (t, *J*=7 Hz, 3 H, CH_3_), 1.3–1.9 (m, 12 H), 2.95 (m, 2 H), 3.16 (m, 2 H), 4.36 (t, *J*=6.4 Hz, 2 H, OC*H*_2_CH_2_), 5.24 (s, 2 H, OCH_2_Ph), 7.09 (dd, *J*=2.7 and 8.9 Hz, 1 H, C2-H), 7.20 (d, *J*=2.6 Hz, 1 H, C4-H), 7.42 (m, 5 H, Ph) and 7.98 ppm (d, *J*=9.4 Hz, 1 H, C1-H); MS (FAB+): *m*/*z* (%) 390.4 (95) [*M*+H]^+^, 319.3 (23) [*M*+H−C_5_H_11_]^+^, 91.1 (100) [Bn^+^]; HRMS-FAB^+^: *m*/*z* [*M*+H]^+^ calcd for C_26_H_32_NO_2_: 390.2433, found: 390.2440. The second fraction that collected upon evaporation gave a light-yellow syrup (380 mg, 976 μmol, 62 %) which was recrystallised from hexane (∼50 mL) to give **20 a** as fine needle-shaped white crystals (189 mg): *R*_f_=0.62 (CHCl_3_), c.f. *R*_f_<0.05 (**17**); mp: 96–98.5 °C; IR (KBr) 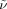
=3000–2840, 1630, 1610, 1590, 1230 cm^−1^; ^1^H NMR (400 MHz, [D_6_]DMSO) *δ*=0.87 (t, *J*=, 7 Hz, 3 H, CH_3_), 1.2–1.62 (m, 9 H), 1.65 (m, 2 H), 2.88 (m, 2 H), 2.98 (m, 2 H), 4.18 (t, *J*=7.4 Hz, 2 H, NCH_2_), 5.29 (s, 2 H, OCH_2_), 6.95 (m, 2 H, C2-H and C4-H), 7.40 (m, 5 H, Ph) and 7.86 ppm (d, *J*=8.9 Hz, 1 H, C1-H); ^13^C NMR (100.4 MHz, CDCl_3_): *δ*=14.03 (q, CH_3_), 22.43 (t), 25.49 (t), 26.15 (t), 26.35 (t), 26.92 (t), 28.22 (t), 29.21 (t), 32.15 (t), 43.18 (t), 70.19 (t, OCH_2_), 99.94 (d), 109.40 (d), 109.53 (d), 114.88 (s), 125.67 (d), 127.03 (d), 127.93 (d), 128.48 (d), 130.45 (s), 136.26 (s), 139.18 (s), 148.09 (s), 159.33 (s) and 161.79 (s); MS (FAB^+^): *m*/*z* (%): 390.4 (100) [*M*+H]^+^, 298.3 (18) [*M*−Bn]^+^, 91.1(39) [Bn]^+^; HRMS-FAB^+^: *m*/*z* [*M*+H]^+^ calcd for C_26_H_32_NO_2_: 390.2433, found: 390.2429; Anal. calcd for C_26_H_31_NO_2_: C 80.17, H 8.02, N, 3.60, found: C 80.0, H 7.99, N 3.57.

**3-Hydroxy-5-pentyl-8,9,10,11-tetrahydro-5*H*-cyclohepta[*c*]quinolin-6(7*H*)-one (20 b).** A solution of **20 a** (405 mg, 1.04 mmol) in absolute EtOH (30 mL) was debenzylated by hydrogenation in similar manner to **18 a** in the presence of Pd/C (10 %, 41 mg). The crude white residue that resulted (235 mg, 785 μmol, 76 %) was recrystallised from CHCl_3_/hexane (3:2) to give **20 b** as white crystals (253 mg, 1.04 mmol, 50 %): *R*_f_=0.52 (CHCl_3_/EtOAc, 8:1), c.f. *R*_f_=0.84 (**20 a**); mp: undefined but all melted by 145 °C; ^1^H NMR (400 MHz, [D_6_]DMSO): *δ*=0.90 (t, *J*∼7 Hz, 3 H, CH_3_), 1.36 (m, 4 H), 1.45 (m, 2 H), 1.58 (m, 4 H), 1.82 (m, 2 H), 2.88 (m, 2 H), 2.97 (m, 2 H), 4.13 (t, *J*=7.3 Hz, 2 H, NCH_2_), 6.71 (dd, *J*=2 and 8.8 Hz, 1 H, C2-H), 6.80 (d, *J*=2.3 Hz, 1 H, C4-H), 7.76 (d, *J*=9 Hz, 1 H, C1-H), 8.32 (CHCl_3_, 0.5 H) and 10.1 ppm (br s, 1 H, ex. with D_2_O, OH); MS (FAB^+^): *m*/*z* (%): 300.3 (100) [*M*+H]^+^, 229.2 (10) [*M*+H−C_5_H_11_]^+^; MS (FAB^−^): *m*/*z* (%): 452.4 (40) [*M*+NBA]^−^, 298.3 (100) [*M*−H]^−^; HRMS-FAB^+^: *m*/*z* [*M*+H]^+^ calcd for C_19_H_26_NO_2_: 300.1964, found: 300.1955.

**6-Oxo-5-pentyl-6,7,8,9,10,11-hexahydro-5*H*-cyclohepta[*c*]quinolin-3-yl sulfamate (20).** Compound **20 b** (178 mg, 595 μmol) in anhydrous DMF (10 mL) was sulfamoylated in a similar manner to **16 a**. The crude light-yellow residue (199 mg) that was obtained was fractionated on silica with CHCl_3_/EtOAc (8:1). The fourth fraction that was collected gave a creamy residue (125 mg, 330 μmol, 56 %) which upon recrystallisation from hot CHCl_3_/hexane (1:2) gave **20** as fine white crystals (97 mg): *R*_f_=0.49 (CHCl_3_/EtOAc, 4:1), c.f. *R*_f_=0.67 (**20 b**); mp: 186–188 °C; IR (KBr) 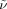
=3650–3000, 3000–2800, 1610, 1560, 1380 cm^−1^; ^1^H NMR (400 MHz, [D_6_]DMSO) *δ*=0.89 (t, *J*=7 Hz, 3 H, CH_3_), 1.37 (m, 4 H), 1.48 (m, 2 H), 1.61 (m, 4 H), 1.84 (m, 2 H), 2.95 (m, 2 H), 3.05 (m, 2 H), 4.21 (t, *J*=7.7 Hz, 2 H, NCH_2_), 7.17 (dd, *J*=2.2 and 8.8 Hz, 1 H, C2-H), 7.36 (d, *J*=2.2 Hz, 1 H, C4-H), 8.05 (d, *J*=8.8 Hz, 1 H, C1-H) and 8.10 ppm (s, 2 H, ex. with D_2_O, OSO_2_NH_2_); MS (FAB^+^): *m*/*z* (%): 379.2 (100) [*M*+H]^+^, 298.2 (27) [*M*−H_2_NSO_2_]^+^; MS (FAB^−^): *m*/*z* (%): 377.2 (100) [*M*−H]^−^, 298.2 (18) [*M*−H_2_NSO_2_]^−^; HRMS-FAB^+^: *m*/*z* [*M*+H]^+^ calcd for C_19_H_27_N_2_O_4_S: 379.1682, found: 379.1702. Anal. calcd for C_19_H_26_N_2_O_4_S: C 60.30, H 6.92, N 7.40, found: C 60.1, H 6.92, N 7.43.

**6-(Pentyloxy)-8,9,10,11-tetrahydro-7*H*-cyclohepta[*c*]quinolin-3-ol (21 b).** A solution of **21 a** (311 mg, 798 μmol) in absolute EtOH (30 mL) was debenzylated by hydrogenation in similar manner to **18 a** in the presence of Pd/C (10 %, 35 mg). The crude light-brown syrup that was obtained was fractionated on silica with CHCl_3_ followed by CHCl_3_/EtOAc (8:1 → 4:1 gradient) to give **21 b** as a light-brown syrup which partially solidified to wax upon standing at room temperature for a few days (160 mg, 534 μmol, 67 %): *R*_f_=0.49 (CHCl_3_/EtOAc, 8:1), c.f. *R*_f_=0.90 (**21 a**); mp: 120 °C; IR (KBr) 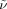
=3700–2500, 3000–2800, 1615, 1590, 1440 cm^−1^; ^1^H NMR (400 MHz, [D_6_]DMSO) *δ*=0.90 (t, *J*∼7 Hz, 3 H, CH_3_), 1.39 (m, 4 H), 1.51 (m, 2 H), 1.59 (m, 2 H), 1.75 (m, 2 H), 1.86 (m, 2 H), 2.93 (m, 2 H), 3.12 (m, 2 H), 4.33 (t, *J*=6.6 Hz, 2 H, OCH_2_), 6.91 (dd, *J*=2.3 and 8.9 Hz, 1 H, C2-H), 6.97 (d, *J*=2.3 Hz, 1 H, C4-H), 7.88 (d, *J*=8.9 Hz, 1 H, C1-H) and 9.81 ppm (br s, 1 H, ex. with D_2_O, OH); MS (FAB^+^): *m*/*z* (%): 300.2 (100) [*M*+H]^+^, 230.1 (30); MS (FAB^−^) *m*/*z* (%) 452.2 (7) [*M*+NBA]^−^, 298.2 (100) [*M*−H]^−^, 228.1(17); HRMS-FAB^+^: *m*/*z* [*M*+H]^+^ calcd for C_19_H_26_NO_2_: 300.1964, found: 300.1962; Anal. calcd for C_19_H_25_NO_2_: C 76.22, H 8.42, N 4.68, found: C 75.9, H 8.50, N, 4.66.

**6-(Pentyloxy)-8,9,10,11-tetrahydro-7*H*-cyclohepta[*c*]quinolin-3-yl sulfamate (21).** Compound **21 b** (140 mg, 468 μmol) in anhydrous DMF (10 mL) was sulfamoylated in a similar manner to **16 a**. The crude light-brown syrup (175 mg) that was obtained was fractionated on silica with EtOAc/hexane (1:3 → 1:2). The second fraction that was collected gave **21** as a yellow syrup that solidified to wax upon standing at room temperature overnight (87 mg, 230 μmol, 49 %): *R*_f_=0.41 (EtOAc/hexane, 1:2), c.f. *R*_f_=0.54 (**21 b**); mp: 103–107 °C; ^1^H NMR (400 MHz, [D_6_]DMSO) *δ*=0.92 (t, *J*=7 Hz, 3 H, CH_3_), 1.41 (m, 4 H), 1.54 (m, 2 H), 1.61 (m, 2 H), 1.78 (m, 2 H), 1.89 (m, 2 H), 3.01 (m, 2 H), 3.22 (m, 2 H), 4.40 (t, *J*=6.5 Hz, 2 H, OCH_2_), 7.30 (dd, *J*=2.3 and 8.9 Hz, 1 H, C2-H), 7.59 (d, *J*=2.3 Hz, 1 H, C4-H), 8.06 (s, 2 H, ex. with D_2_O, OSO_2_NH_2_) and 8.17 ppm (d, *J*=9 Hz, 1 H, C1-H); MS (FAB^+^): *m*/*z* (%): 379.2 (100) [*M*+H]^+^, 300.2 (5) [*M*+H−HNSO_2_]^+^; MS (FAB^−^): *m*/*z* (%): 377.1 (100) [*M*−H]^−^, 298.2 (11) [*M*−H_2_NSO_2_]^−^, 77.9 (52); HRMS-FAB^+^: *m*/*z* [*M*+H]^+^ calcd for C_19_H_27_N_2_O_4_S: 379.1692, found: 379.1691; Anal. calcd for C_19_H_26_N_2_O_4_S: C 60.30, H 6.92, N 7.40, found: C 60.5, H 7.05, N 7.34.

**3-(Benzyloxy)-5-(3-phenylpropyl)-8,9,10,11-tetrahydro-5*H*-cyclohepta[*c*]quinolin-6(7*H*)-one (22 a) and 3-(benzyloxy)-6-(3-phenylpropoxy)-8,9,10,11-tetrahydro-7*H*-cyclohepta[*c*]quino-line (23 a).** NaH (60 % in mineral oil, 65 mg, 1.62 mmol) was added to a solution of **17** (500 mg, 1.57 mmol) in DMF (40 mL) at room temperature cautiously followed by 1-bromo-3-phenylpropane (0.25 mL, 1.65 mmol) 15 min later after the evolution of H_2_ had ceased. The reaction mixture was heated at 100 °C for 1 h and then concentrated in vacuo after cooling to room temperature. The crude material that was obtained was dissolved in EtOAc (100 mL) and the resulting mixture was washed with brine (4×50 mL), dried (MgSO_4_), filtered and evaporated to give a yellow syrup which was fractionated on silica eluting first with CHCl_3_/hexane (2:1 → 4:1 gradient), then CHCl_3_ followed by CHCl_3_/EtOAc (2:1 → 1:2 gradient). The first fraction that was collected upon evaporation gave **23 a** as a light-yellow syrup (287 mg, 656 μmol, 42 %): *R*_f_=0.71 (CHCl_3_/hexane, 2:1), c.f. *R*_f_<0.1 (**17**); ^1^H NMR (400 MHz, [D_6_]DMSO): *δ*=1.57 (m, 4 H), 2.09 (quintet, *J*=7 Hz, 2 H, OCH_2_C*H*_2_CH_2_Ph), 2.77 (t, 2 H, CH_2_C*H*_2_Ph), 2.98 (m, 2 H), 3.16 (m, 2 H), 4.35 (t, *J*=6.3 Hz, 2 H, OC*H*_2_CH_2_), 5.23 (s, 2 H, OCH_2_Ph), 7.09 (dd, *J*=2.5 and 9 Hz, 1 H, C2-H), 7.34 (m, 11 H, C4-H and 2×Ar) and 7.98 ppm (d, *J*=9 Hz, 1 H, C1-H); MS (FAB^+^): *m*/*z* (%): 438.4 (20) [*M*+H]^+^, 319.3 (14) [*M*+H-CH_2_CH_2_CH_2_Ph]^+^, 91.1 (45) [Bn]^+^, 73.1(100); HRMS-FAB^+^: *m*/*z* [*M*+H]^+^ calcd for C_30_H_32_NO_2_: 438.2433, found: 438.2438. The second fraction that was collected upon evaporation gave a clear syrup (373 mg, 852 μmol, 55 %) that was recrystallised from EtOAc/hexane (1:15, ∼32 mL) to give **22 a** as light-beige rod-shaped crystals (240 mg): *R*_f_=0.38 (CHCl_3_/hexane, 2:1), c.f. *R*_f_<0.1 (**17**); mp: 120–122 °C; ^1^H NMR (400 MHz, [D_6_]DMSO) *δ*=1.45 (m, 2 H), 1.56 (m, 2 H), 1.83 (m, 4 H), 2.71 (t, *J*=7.4 Hz, 2 H, CH_2_C*H*_2_Ph), 2.89 (m, 2 H), 2.98 (m, 2 H), 4.22 (t, *J*=7.6 Hz, 2 H, NCH_2_), 5.17 (s, 2 H, OCH_2_Ph), 6.83 (d, *J*=2.3 Hz, 1 H, C4-H), 6.93 (dd, *J*=2.3 and 8 Hz, 1 H, C2-H), 7.34 (m, 10 H, Ar) and 7.86 ppm (d, *J*=9 Hz, 1 H, C1-H); MS (FAB^+^): *m*/*z* (%): 438.4 (100) [*M*+H]^+^, 346.3 (20) [*M*−Bn]^+^, 91.1 (62) [Bn]^+^; HRMS-FAB^+^: *m*/*z* [*M*+H]^+^ calcd for C_30_H_32_NO_2_: 438.2433, found: 438.2423. Anal. calcd for C_30_H_31_NO_2_: C 82.35, H 7.14, N, 3.20, found: C 82.7, H 7.14, N 3.43.

**3-Hydroxy-5-(3-phenylpropyl)-8,9,10,11-tetrahydro-5*H*-cyclohepta[*c*]quinolin-6(7*H*)-one (22 b).** A solution of **22 a** (300 mg, 686 μmol) in absolute EtOH (30 mL) was debenzylated by hydrogenation in similar manner to **18 a** in the presence of Pd/C (10 %, 60 mg). The crude solid that resulted (205 mg, 590 μmol, 86 %) was recrystallised from toluene/hexane (8:3) to give **22 b** as creamy crystals (150 mg): *R*_f_=0.68 (CHCl_3_/EtOAc, 4:1), c.f. *R*_f_=0.91 (**22 a**); mp: 182–186 °C; ^1^H NMR (400 MHz, [D_6_]DMSO): *δ*=1.45 (m, 2 H), 1.56 (m, 2 H), 1.81 (m, 2 H), 1.90 (quintet, *J*=7.8 Hz, 2 H, NCH_2_C*H*_2_CH_2_Ph), 2.73 (t, *J*=7.8 Hz, 2 H, CH_2_Ph), 2.88 (m, 2 H), 2.96 (m, 2 H), 4.18 (t, *J*=7 Hz, 2 H, NCH_2_), 6.72 (dd, *J*=2.3 and 8.9 Hz, 1 H, C2-H), 6.78 (d, *J*=2 Hz, 1 H, C4-H), 7.25 (m, 5 H, Ph), 7.76 (d, *J*=8.9 Hz, 1 H, C1-H) and 10.1 (br s, 1 H, ex. with D_2_O, OH); MS (FAB^+^): *m*/*z* (%): 348.3 (100) [*M*+H]^+^, 243.2 (18); MS (FAB^−^): *m*/*z* (%): 500.3(47) [*M*+NBA]^−^, 346.3 (100) [*M*−H]^−^; HRMS-FAB^+^: *m*/*z* [*M*+H]^+^ calcd for C_23_H_26_NO_2_: 348.1964, found: 348.1964.

**6-Oxo-5-(3-phenylpropyl)-6,7,8,9,10,11-hexahydro-5*H*-cyclohepta[*c*]quinolin-3-yl sulfamate (22).** Compound **22 b** (100 mg, 288 μmol) in anhydrous DMF (10 mL) was sulfamoylated in a similar manner to **16 a**. The crude light-yellow residue (122 mg) obtained was fractionated on silica with CHCl_3_/EtOAc (8:1). The second fraction that was collected gave a light-yellow syrup that solidified on standing overnight to give **22** as a white solid (65 mg, 152 μmol, 53 %). Recrystallisation from hot CHCl_3_/hexane (5:4) gave **22** as fine white crystals (39 mg): *R*_f_=0.23 (CHCl_3_/EtOAc, 8:1), c.f. *R*_f_=0.43 (**22 b**); mp: 176–179 °C; IR (KBr) 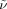
=3650–3000, 3000–2800, 1610, 1560, 1380, 1190 cm^−1^; ^1^H NMR (400 MHz, [D_6_]DMSO) *δ*=1.45 (m, 2 H), 1.56 (m, 2 H), 1.83 (m, 2 H), 1.91 (m, 2 H), 2.71 (t, *J*=7.7 Hz, 2 H, CH_2_Ph), 2.92 (m, 2 H), 3.03 (m, 2 H), 4.24 (t, *J*=7.7 Hz, 2 H, CH_2_N), 7.22 (m, 6 H, C2-H and Ar), 7.35 (d, *J*=2.2 Hz, 1 H, C4-H), 8.03 (d, *J*=8.8 Hz, 1 H, C1-H) and 8.08 ppm (br s, 2 H, ex. with D_2_O, OSO_2_NH_2_); MS (FAB^+^): *m*/*z* (%): 427.2 (100) [*M*+H]^+^, 346.2 (25) [*M*−H_2_NSO_2_]^+^; MS (FAB^−^): *m*/*z* (%): 425.1(100) [*M*−H]^−^, 346.2 (19) [*M*−H_2_NSO_2_]^−^; HRMS-FAB^+^: *m*/*z* [*M*+H]^+^ calcd for C_23_H_27_N_2_O_4_S: 427.1620, found 427.1695. Anal. calcd for C_23_H_26_N_2_O_4_S: C 64.77, H 6.14, N 6.57, found: C 64.2, H 6.13, N 6.65.

**6-(3-Phenylpropoxy)-8,9,10,11-tetrahydro-7*H*-cyclohepta[*c*]quinolin-3-ol (23 b).** A solution of **23 a** (255 mg, 583 μmol) in a mixture of absolute EtOH (30 mL) and THF (10 mL) was debenzylated by hydrogenation in similar manner to **18 a** in the presence of Pd/C (10 %, 51 mg). The crude yellow syrup that was obtained solidified on standing overnight to give **23 b** as a yellow wax (182 mg, 524 μmol, 90 %): *R*_f_=0.61 (CHCl_3_/EtOAc, 4:1), c.f. *R*_f_=0.88 (**23 a**); mp: ∼135 °C; IR (KBr) 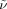
=3700–2500, 3000–2800, 1615, 1590, 1420, 1330, 1200 cm^−1^; ^1^H NMR (400 MHz, [D_6_]DMSO) *δ*=1.57 (m, 4 H), 1.86 (m, 2 H), 2.08 (m, 2 H, CH_2_C*H*_2_CH_2_Ph), 2.77 (t, *J*=7.5 Hz, 2 H, CH_2_Ph),2.95 (m, 2 H), 3.13 (m, 2 H), 4.33 (t, *J*=6.4 Hz, 2 H, OCH_2_), 6.91 (dd, *J*=2.7 and 8.9 Hz, 1 H, C2-H), 6.95 (d, *J*=2.3 Hz, 1 H, C4-H), 7.25 (m, 5 H, Ar), 7.88 (d, *J*=8.9 Hz, 1 H, C1-H) and 9.82 (br s, 1 H, ex. with D_2_O, OH); MS (FAB^+^): *m*/*z* (%): 348.3 (100) [*M*+H]^+^, 229.3 (40) [*M*+H−CH_2_CH_2_CH_2_Ph]^+^; MS (FAB^−^): *m*/*z* (%): 346.3 (100) [*M*−H]^−^, 275.2 (40), 181.2 (50); HRMS-FAB^+^: *m*/*z* [*M*+H]^+^ calcd for C_23_H_26_NO_2_: 348.1964, found: 348.1969.

**6-(3-Phenylpropoxy)-8,9,10,11-tetrahydro-7*H*-cyclohepta[*c*]quinolin-3-yl sulfamate (23).** Compound **23 b** (135 mg, 389 μmol) in anhydrous DMF (10 mL) was sulfamoylated in a similar manner to **16 a**. The crude light-brown syrup (175 mg) that obtained was fractionated on silica with EtOAc/hexane (1:3 → 1:2). The second fraction that was collected gave **23** as a yellow syrup that solidified to wax upon standing at room temperature overnight (87 mg, 230 μmol, 49 %): *R*_f_=0.41 (EtOAc/hexane, 1:2), c.f. *R*_f_=0.54 (**23 b**); mp: 103–107 °C; ^1^H NMR (400 MHz, [D_6_]DMSO): *δ*=0.92 (t, *J*=7 Hz, 3 H, CH_3_), 1.41 (m, 4 H), 1.54 (m, 2 H), 1.61 (m, 2 H), 1.78 (m, 2 H), 1.89 (m, 2 H), 3.01 (m, 2 H), 3.22 (m, 2 H), 4.40 (t, *J*=6.5 Hz, 2 H, OCH_2_), 7.30 (dd, *J*=2.3 and 8.9 Hz, 1 H, C2-H), 7.59 (d, *J*=2.3 Hz, 1 H, C4-H), 8.06 (s, 2 H, ex. with D_2_O, OSO_2_NH_2_) and 8.17 ppm (d, *J*=9 Hz, 1 H, C1-H); MS (FAB^+^): *m*/*z* (%): 379.2 (100) [*M*+H]^+^, 300.2 (5) [*M*+H−HNSO_2_]^+^; MS (FAB^−^): *m*/*z* (%): 377.1 (100) [*M*−H]^−^, 298.2 (11) [*M*−H_2_NSO_2_]^−^, 77.9 (52); HRMS-FAB^+^: *m*/*z* [*M*+H]^+^ calcd for C_19_H_27_N_2_O_4_S: 379.1692, found: 379.1691. Anal. calcd for C_19_H_26_N_2_O_4_S: C 60.30, H 6.92, N 7.40, found: C 60.5, H 7.05, N 7.34.

**3-Amino-8,9,10,11-tetrahydro-5*H*-cyclohepta[*c*]quinolin-6(7*H*)-one (24 a).** A mixture of 1,3-phenylenediamine (5.0 g, 46.22 mmol) and methyl 2-oxo-1-cycloheptane carboxylate (7.9 g, 46.22 mmol) was heated at 150 °C overnight. The yellow sludge that formed was cooled to room temperature and diluted with Et_2_O to give a yellow suspension which was filtered. The beige precipitate collected (3.91 g) was recrystallised from hot *i*PrOH to give **24 a** as a wool-like fluff (1.59 g, 6.98 mmol, 15 %): mp: 290–300 °C (dec); IR (KBr) 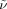
=3460, 3360, 2920, 2850, 1650, 1620 cm^−1^; ^1^H NMR (400 MHz, [D_6_]DMSO): *δ*=1.42 (m, 2 H), 1.54 (m, 2 H), 1.80 (m, 2 H), 2.78 (m, 2 H, C7-H_2_), 2.87 (m, 2 H, C11-H_2_), 5.63 (br s, ∼2 H, ex. with D_2_O, NH_2_), 6.37 (d, *J*=2.1 Hz, 1 H, C4-H_2_), 6.64 (dd, *J*=2.1 and 8.9 Hz, 1 H, C2-H_2_), 7.46 (d, *J*=8.8 Hz, 1 H, C1-H_2_) and 11.2 (br s, 1 H, exchanged with D_2_O, CONH). Anal. calcd for C_14_H_16_N_2_O: C 73.66, H 7.06, N 12.27, found: C 73.7, H 7.21, N, 12.1.

**6-Oxo-6,7,8,9,10,11-hexahydro-5*H*-cyclohepta[*c*]quinolin-3-ylsulfamide (24).** To a solution of **24 a** (300 mg; 1.31 mmol) and 2,6-di-*tert*-butyl-4-methylpyridine (270 mg, 1.31 mmol) in anhydrous DMF (20 mL) at 0 °C under N_2_ was added sulfamoyl chloride (∼0.69 m in toluene,[19] ∼3–5 equiv, evaporated down to ∼1 mL prior to addition). After stirring at room temperature under N_2_ overnight, the reaction mixture was diluted with EtOAc (∼150 mL) and the organic layer washed with brine (5×100 mL). After drying with MgSO_4_ and filtering, the filtrate was evaporated, during which time the precipitation of **24** occurred. Collection and air drying of the precipitate gave **24** as a white powder (113 mg; 28 %): *R*_f_=0.46 (CHCl_3_/EtOAc, 4:1); mp: 183–185 °C; IR (KBr) 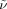
=3700–2700, 3360, 3280, 2920, 2840, 1630, 1340, 1160 cm^−1^; ^1^H NMR (270 MHz; [D_6_]DMSO): *δ*=1.44 (m, 2 H), 1.56 (m, 2 H), 1.83 (m, 2 H), 2.81 (m, 2 H, C7-CH_2_), 2.94 (m, 2 H, C11-CH_2_), 7.03 (dd, *J*=2.2 and 8.8 Hz, 1 H, C2-H), 7.10 (d, *J*=2.1 Hz, 1 H, C4-H), 7.13 (br s, 2 H, ex. with D_2_O, *H*_2_NSO_2_NH), 7.73 (d, *J*=9 Hz, 1 H, C1-H), 9.81 (s, 1 H, ex. with D_2_O, H_2_NSO_2_N*H*) and 11.52 (s, 1 H, ex. with D_2_O, CONH); MS (FAB^+^): *m*/*z* (%): 308.1 (100) [*M*+H]^+^; MS (FAB^−^): *m*/*z* (%): 306.2 (100) [*M*−H]^−^, HRMS-FAB^+^: *m*/*z* [*M*+H]^+^ calcd for C_14_H_18_N_3_O_3_S: 308.1069, found: 308.1055; Anal. calcd for C_14_H_17_N_3_O_3_S: C 54.71, H 5.57, N 13.67, found: C 54.5, H 5.60, N 13.5.
