# Supplementary material for: Structure–Activity Relationship for the First-in-Class Clinical Steroid Sulfatase Inhibitor Irosustat (STX64, BN83495)
Source: ChemMedChem. 2011 Aug 25;6(11):2019–34. doi: 10.1002/cmdc.201100288 (PMC3262147; doi:10.1002/cmdc.201100288)

CHEMMEDCHEM
Chemistry & Drug Discovery

Supporting Information

© Copyright Wiley-VCH Verlag GmbH & Co. KGaA, 69451 Weinheim, 2011

Structure–Activity Relationship for the First-in-Class Clinical Steroid Sulfatase Inhibitor Irosustat (STX64, BN83495)

L. W. Lawrence Woo,[a] Dharshini Ganeshapillai,[a] Mark P. Thomas,[a] Oliver B. Sutcliffe,[a] Bindu Malini,[b] Mary F. Mahon,[c] Atul Purohit,[b] and Barry V. L. Potter*[a]

cmdc_201100288_sm_miscellaneous_information.pdf

checkCIF/PLATON report

You have not supplied any structure factors. As a result the full set of tests cannot be run.

No syntax errors found.   CIF dictionary   Interpreting this report

Datablock: Compound-1-k00bvlp4

```
Bond precision:  C-C = 0.0053 A          Wavelength=0.71073

Cell:           a=10.4000(13)    b=11.1750(11)     c=11.9180(13)
                alpha=90         beta=99.585(6)    gamma=90
Temperature:    150 K

                Calculated                 Reported
Volume          1365.8(3)                  1365.8(3)
Space group     P 21/a                     P21/a
Hall group      -P 2yab                    ?
Moiety formula  C14 H15 N O5 S             ?
Sum formula     C14 H15 N O5 S             C14 H15 N O5 S
Mr              309.34                     309.33
Dx,g cm-3       1.504                      1.504
Z               4                          4
Mu (mm-1)       0.259                      0.259
F000            648.0                      648.0
F000'           648.84
h,k,lmax        13,14,15                   13,14,15
Nref            3147                       3104
Tmin,Tmax       0.969,0.979                0.909,0.911
Tmin'           0.937

Correction method= MULTI-SCAN

Data completeness= 0.986          Theta(max)= 27.530

R(reflections)= 0.0847( 2131)     wR2(reflections)= 0.2346( 3104)

S = 1.041                   Npar= 199
```

The following ALERTS were generated. Each ALERT has the format
test-name_ALERT_alert-type_alert-level.
Click on the hyperlinks for more details of the test.

Alert level B

```
RINTA01_ALERT_3_B  The value of Rint is greater than 0.18
            Rint given   0.184
PLAT020_ALERT_3_B The value of Rint is greater than 0.12 .........       0.18
```

Alert level C

```
PLAT340_ALERT_3_C Low Bond Precision on  C-C Bonds (x 1000) Ang ..          5
```

Alert level G

```
PLAT002_ALERT_2_G Number of Distance or Angle Restraints on AtSite          3
PLAT072_ALERT_2_G SHELXL First  Parameter in WGHT Unusually Large.       0.13
PLAT128_ALERT_4_G Alternate Setting of Space-group P21/c   .......      P21/a
PLAT180_ALERT_4_G Check Cell Rounding: # of Values Ending with 0 =          3
PLAT860_ALERT_3_G Note: Number of Least-Squares Restraints .......          2
```

0 **ALERT level A** = Most likely a serious problem - resolve or explain
2 **ALERT level B** = A potentially serious problem, consider carefully
1 **ALERT level C** = Check. Ensure it is not caused by an omission or oversight
5 **ALERT level G** = General information/check it is not something unexpected

0 ALERT type 1 CIF construction/syntax error, inconsistent or missing data
2 ALERT type 2 Indicator that the structure model may be wrong or deficient
4 ALERT type 3 Indicator that the structure quality may be low
2 ALERT type 4 Improvement, methodology, query or suggestion
0 ALERT type 5 Informative message, check

Datablock: Compound-15-k01pharm2

Bond precision: C-C = 0.0027 A   Wavelength=0.71073

Cell:	a=8.2170(2)	b=8.6730(2)	c=13.0540(4)
	alpha=104.432(1)	beta=97.598(1)	gamma=108.727(1)
Temperature:	150 K		

	Calculated	Reported
Volume	830.09(4)	830.09(4)
Space group	P -1	P-1
Hall group	-P 1	?
Moiety formula	C17 H20 N2 O5 S	?
Sum formula	C17 H20 N2 O5 S	C17 H20 N2 O5 S
Mr	364.42	364.41
Dx,g cm-3	1.458	1.458
Z	2	2
Mu (mm-1)	0.227	0.227
F000	384.0	384.0
F000’	384.43	
h,k,lmax	10,11,16	10,11,16
Nref	3809	3788
Tmin,Tmax	0.965,0.978	0.891,0.982
Tmin’	0.945	

Correction method= MULTI-SCAN

```
Data completeness= 0.994          Theta(max)= 27.480

R(reflections)= 0.0451( 2637)     wR2(reflections)= 0.1221( 3788)

S = 0.981                  Npar= 229
```

The following ALERTS were generated. Each ALERT has the format
test-name_ALERT_alert-type_alert-level.
Click on the hyperlinks for more details of the test.

Alert level C

```
PLAT230_ALERT_2_C Hirshfeld Test Diff for    S1     --  O2      ..       6.22 su
```

Alert level G

```
PLAT154_ALERT_1_G The su's on the Cell Angles are Equal  (x 10000)        100 Deg.
PLAT180_ALERT_4_G Check Cell Rounding: # of Values Ending with 0 =          3
PLAT380_ALERT_4_G Check Incorrectly? Oriented X(sp2)-Methyl Moiety        C18
PLAT808_ALERT_5_G No Parseable SHELXL Style Weighting Scheme Found          !
```

---

0 **ALERT level A** = Most likely a serious problem - resolve or explain
0 **ALERT level B** = A potentially serious problem, consider carefully
1 **ALERT level C** = Check. Ensure it is not caused by an omission or oversight
4 **ALERT level G** = General information/check it is not something unexpected

1 ALERT type 1 CIF construction/syntax error, inconsistent or missing data
1 ALERT type 2 Indicator that the structure model may be wrong or deficient
0 ALERT type 3 Indicator that the structure quality may be low
2 ALERT type 4 Improvement, methodology, query or suggestion
1 ALERT type 5 Informative message, check

It is advisable to attempt to resolve as many as possible of the alerts in all categories. Often the minor alerts point to easily fixed oversights, errors and omissions in your CIF or refinement strategy, so attention to these fine details can be worthwhile. In order to resolve some of the more serious problems it may be necessary to carry out additional measurements or structure refinements. However, the purpose of your study may justify the reported deviations and the more serious of these should normally be commented upon in the discussion or experimental section of a paper or in the "special_details" fields of the CIF. checkCIF was carefully designed to identify outliers and unusual parameters, but every test has its limitations and alerts that are not important in a particular case may appear. Conversely, the absence of alerts does not guarantee there are no aspects of the results needing attention. It is up to the individual to critically assess their own results and, if necessary, seek expert advice.

Publication of your CIF in IUCr journals

A basic structural check has been run on your CIF. These basic checks will be run on all CIFs submitted for publication in IUCr journals (*Acta Crystallographica*, *Journal of Applied Crystallography*, *Journal of Synchrotron Radiation*); however, if you intend to submit to *Acta Crystallographica Section C* or *E*, you should make sure that full publication checks are run on the final version of your CIF prior to submission.

Publication of your CIF in other journals

Please refer to the *Notes for Authors* of the relevant journal for any special instructions relating to CIF submission.

PLATON version of 24/03/2011; check.def file version of 16/03/2011

Datablock Compound-1-k00bvlp4 - ellipsoid plot

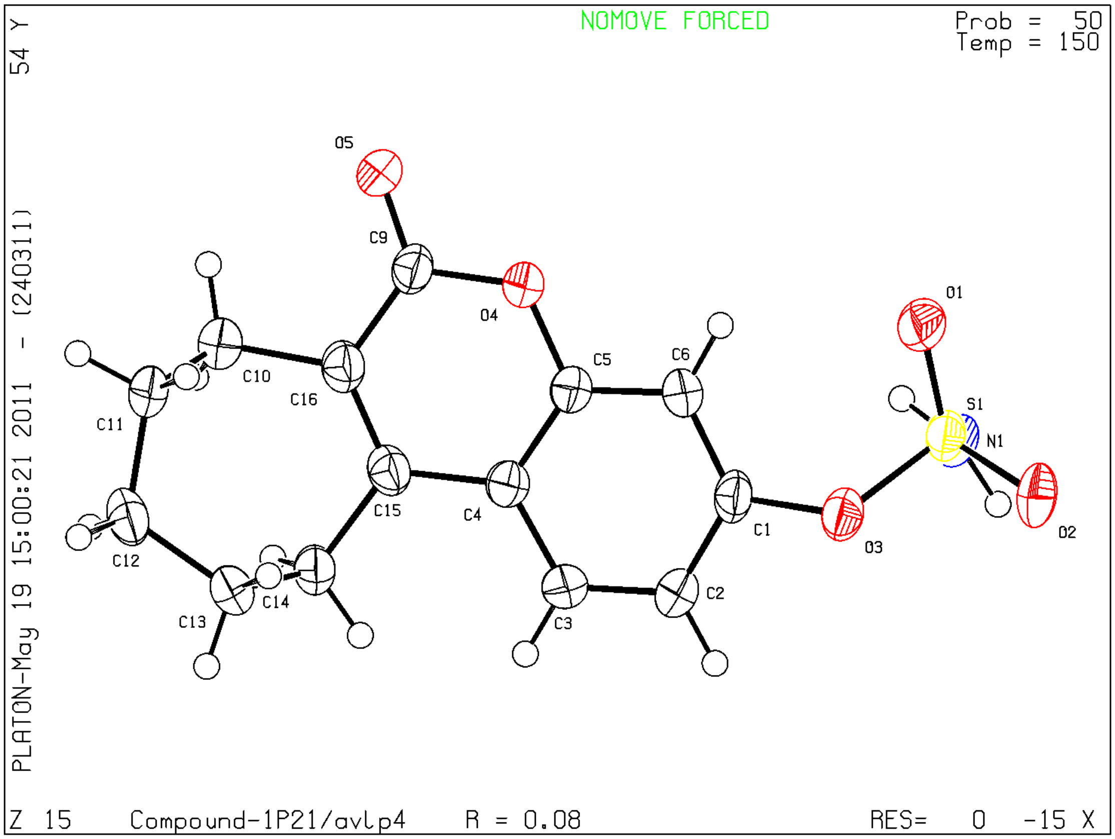

Datablock Compound-15-k01pharm2 - ellipsoid plot

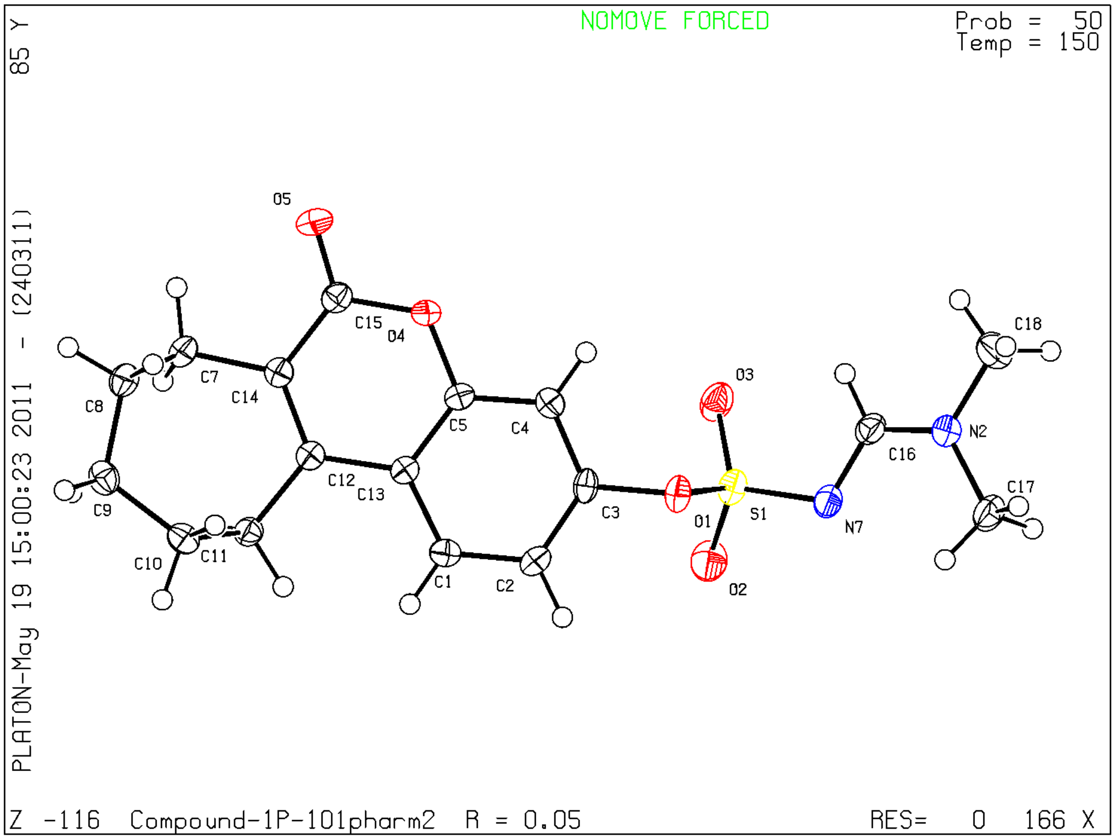

Supplement: Supplementary file 1 [file cmdc0006-2019-SD1.pdf]
